# The longevity response to warm temperature is neurally controlled via the regulation of collagen genes

**DOI:** 10.1111/acel.13815

**Published:** 2023-03-09

**Authors:** Sankara Naynar Palani, Durai Sellegounder, Phillip Wibisono, Yiyong Liu

**Affiliations:** ^1^ Department of Translational Medicine and Physiology, Elson S. Floyd College of Medicine Washington State University Spokane Washington USA; ^2^ Genomics Core Washington State University Spokane Washington USA

**Keywords:** aging, *C. elegans*, G protein‐coupled receptor, gene expression, lifespan, longevity, metabolic rate, neuroscience, NPR‐8, signal transduction

## Abstract

Studies in diverse species have associated higher temperatures with shorter lifespan and lower temperatures with longer lifespan. These inverse effects of temperature on longevity are traditionally explained using the rate of living theory, which posits that higher temperatures increase chemical reaction rates, thus speeding up the aging process. Recent studies have identified specific molecules and cells that affect the longevity response to temperature, indicating that this response is regulated, not simply thermodynamic. Here, we demonstrate that in *Caenorhabditis elegans*, functional loss of NPR‐8, a G protein‐coupled receptor related to mammalian neuropeptide Y receptors, increases worm lifespan at 25°C but not at 20°C or 15°C, and that the lifespan extension at 25°C is regulated by the NPR‐8‐expressing AWB and AWC chemosensory neurons as well as AFD thermosensory neurons. Integrative transcriptomic analyses revealed that both warm temperature and old age profoundly alter gene expression and that genes involved in the metabolic and biosynthetic processes increase expression at 25°C relative to 20°C, indicating elevated metabolism at warm temperature. These data demonstrate that the temperature‐induced longevity response is neurally regulated and also provide a partial molecular basis for the rate of living theory, suggesting that these two views are not mutually exclusive. Genetic manipulation and functional assays further uncovered that the NPR‐8‐dependent longevity response to warm temperature is achieved by regulating the expression of a subset of collagen genes. As increased collagen expression is a common feature of many lifespan‐extending interventions and enhanced stress resistance, collagen expression could be critical for healthy aging.

## Graphical abstract

Studies in diverse species have associated higher temperatures with shorter lifespan and lower temperatures with longer lifespan, but the underlying mechanisms remain unclear. We discovered that functional loss of the neuronal GPCR NPR‐8 extends *C. elegans* lifespan at warm temperature. This longevity response to warm temperature is regulated by the amphid sensory neurons AWB and AWC as well as AFD thermosensory neurons and is achieved by controlling the expression of a subset of collagen genes.

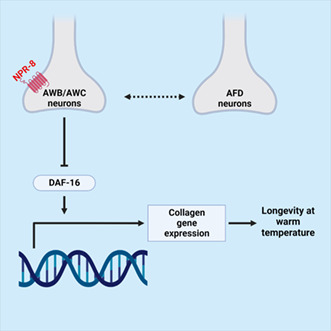

## 1 INTRODUCTION

Studies in diverse species have associated lower temperatures with longer lifespan and higher temperatures with shorter lifespan, aside from extreme temperatures that could endanger organism survival (Keil et al., 2015). Most early studies on the longevity response to temperature attempted to interpret the inverse relationship using the rate of living theory, which posits that higher temperatures increase chemical reaction rates, thus speeding up the aging process, whereas lower temperatures do just the opposite (Demetrius, 2013). At the core of this theory is applying the second law of thermodynamics to living organisms, speculating that aging results from increased molecular disorder, and that higher temperatures enhance thermodynamic entropy, thus inducing a faster rate of aging (Demetrius, 2013). Macromolecular disorder or damage indeed accumulates with age, mainly inflicted by mitochondria‐produced reactive oxygen species (ROS; Sohal et al., 2002). However, many studies have found no connections between longevity and antioxidant treatments or antioxidant overexpression, and some have even linked higher levels of oxidative damage with longer lifespan (Lapointe & Hekimi, 2010). Thus, whether ROS or mitochondrial oxidative stress is causal to the aging process remains unresolved, and the molecular basis for the rate of living theory is unsubstantiated.

Although temperatures could alter the accumulation of molecular damage, the view that temperatures thermodynamically change lifespan is overly simplistic. Indeed, recent studies have identified specific cells, cellular molecules, and signaling pathways that are involved in the longevity response to temperature, indicating that this response is a regulated process, not simply thermodynamic (Keil et al., 2015; Kim et al., 2020). In *C. elegans*, the cold‐sensitive TRP channel TRPA‐1 can detect temperature drops in the environment and extend lifespan by inducing calcium influx into the cell and activating protein kinase C, which, in turn, activates the pro‐longevity transcription factor DAF‐16/FOXO (Xiao et al., 2013). Interestingly, TRPA‐1 also mediates lifespan shortening when worms are subjected to low‐temperature treatment during the larval stage (Zhang et al., 2015). Moreover, thermosensory AFD neurons and AIY interneurons were found to be required for maintaining *C. elegans* lifespan at warm temperature (25°C) through the DAF‐9/DAF‐12 steroid‐signaling pathway (Lee & Kenyon, 2009). Such neural regulation was controlled by the worm cyclic AMP‐responsive element‐binding protein (CREB) transcription factor (Chen et al., 2016). DAF‐41, the *C. elegans* homolog of co‐chaperone P23, which has a possible role in protein folding, is also required for the normal longevity responses to both cold and warm temperatures, indicating a potential role of proteostasis in these responses (Horikawa et al., 2015). Several factors involved in lipid metabolism pathways, including MDT‐15/Mediator 15 (Lee, An, et al., 2019), PAQR‐2 (Chen et al., 2019) and prostaglandin (Lee, Noormohammadi, et al., 2019), also modulate longevity in response to low temperatures. Measuring the lifespans of a diverse array of short‐ or long‐lived mutant worms at different temperatures revealed that temperature‐induced lifespan changes are genetically controlled by temperature‐specific gene regulation (Miller et al., 2017). These studies suggest that the longevity response to temperature is a complex and highly regulated process, and that integrative research is needed to define the relationships between temperature, aging mechanisms, and longevity.

Here, using the *C. elegans* model, we demonstrate that functional loss of NPR‐8, a neuronal G protein‐coupled receptor (GPCR) related to mammalian neuropeptide Y receptors, extends worm lifespan at 25°C but not at 20°C or 15°C, and that the extension in lifespan at 25°C is regulated by the NPR‐8‐expressing amphid chemosensory neurons AWB and AWC as well as AFD thermosensory neurons, indicating that the nervous system governs longevity in response to higher temperature. Integrative RNA sequencing analysis to examine the relationships between temperature, aging, and NPR‐8 revealed that both warm temperature and old age profoundly alter gene expression. Genetic manipulation and functional assays further uncovered that NPR‐8‐regulated collagen expression contributes to the extended lifespan of *npr‐8* mutant animals at 25°C and is also required for maintaining the lifespan of wild‐type animals. Since elevated collagen expression is a common feature of many lifespan‐extending interventions and enhanced stress resistance to various environmental stimuli (Ewald et al., 2015; Sellegounder et al., 2019), collagen expression could promote healthy aging.

## 2 RESULTS

### 2.1 Functional loss of NPR‐8 extends *C. elegans* lifespan at warm temperature

**FIGURE 1:**
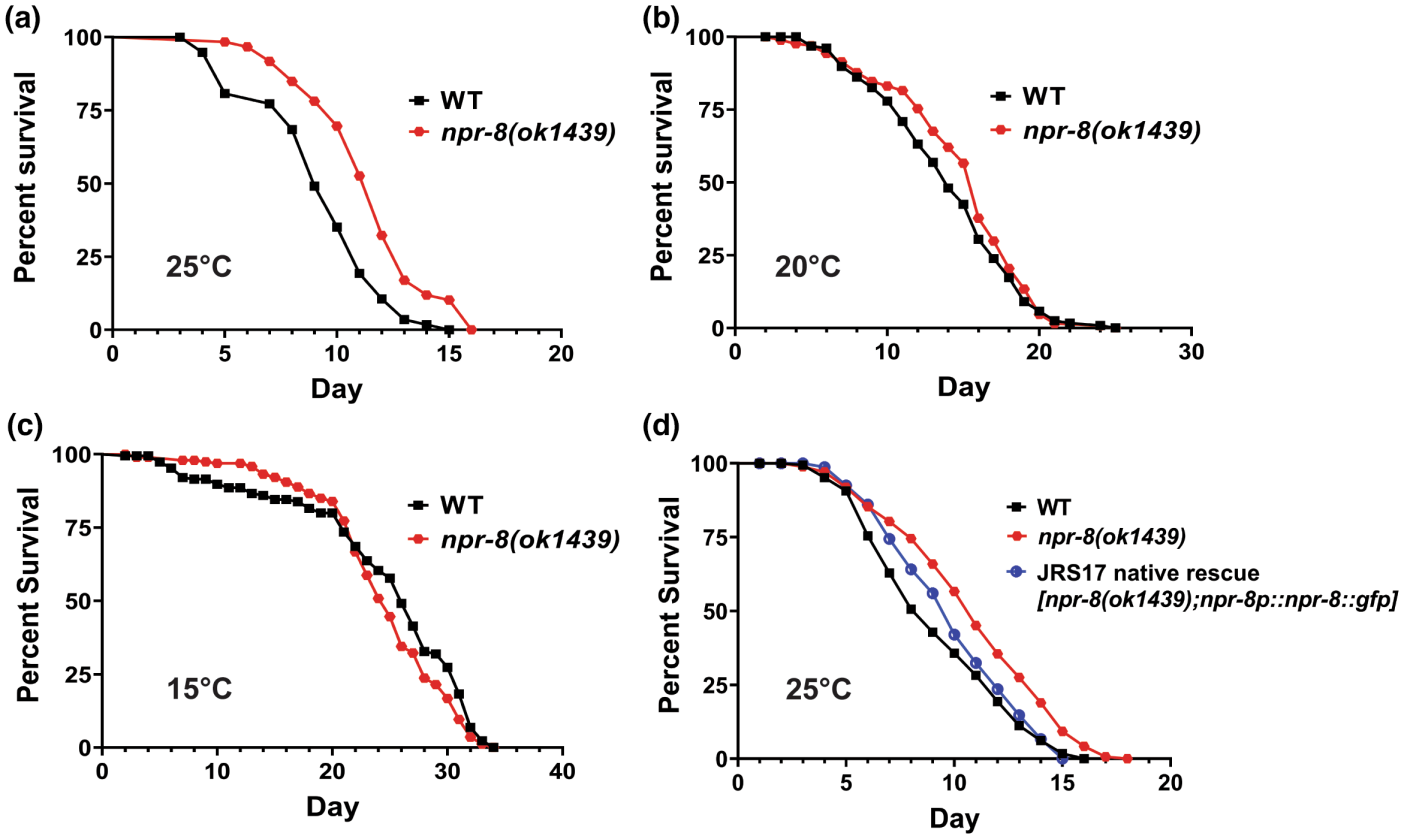
Functional loss of NPR‐8 extends lifespan at 25°C but not at 20°C or 15°C. WT and *npr‐8(ok1439)* animals were grown on *E. coli* strain OP50 at 25°C (a), 20°C (b), or 15°C (c), and scored for survival over time. The graphs are the combined results of three independent experiments. Each experiment included *n* = 60 adult animals per strain. *p*‐Values represent the significance level of the mutants relative to the WT, *p* < 0.0001 in (a), *p* = 0.1640 in (b), and *p* = 0.1126 in (c). (d) WT, *npr‐8(ok1439)*, JRS17 (*npr‐8* expression restored in *npr‐8(ok1439)* under its native promoter, genotype: *npr‐8(ok1439);npr‐8p::npr‐8::gfp*) animals were grown on *E. coli* strain OP50 at 25°C and scored for survival over time. The graphs are the combined results of three independent experiments. Each experiment included *n* = 60 adult animals per strain. *p*‐values are relative to *npr‐8(ok1439)*: WT, *p* < 0.0001; JRS17, *p* < 0.0001.

Three temperatures (15, 20, and 25°C) are routinely used for *C. elegans* propagation in the laboratory, and their inverse effects on lifespan have been well documented, (i.e., an approximately 75% increase in lifespan occurs with a 5°C drop in temperature) (Van Voorhies & Ward, 1999). During our work on neural regulation of *C. elegans* defense against pathogen infection (Sellegounder et al., 2019), we unexpectedly observed that under noninfectious conditions, *C. elegans* lacking functional NPR‐8 (*npr‐8(ok1439)* animals) lived longer than wild‐type *N2* animals at 25°C, whereas the mutant and wild‐type animals had the same lifespan at 20°C or 15°C (Figure 1a–c, also see Table S1 that summarizes the median lifespan and the percentage of increase in median lifespan relative to controls for all lifespan assays performed in this study). To determine whether the observed longevity phenotype at the warm temperature of 25°C was due to the deletion mutation in *npr‐8*, we performed lifespan assays at 25°C using the transgenic strain JRS17 in which *npr‐8* is re‐expressed under its native promoter in *npr‐8(ok1439)* animals (Sellegounder et al., 2019). Re‐expression of NPR‐8 significantly decreased the extended lifespan of the *npr‐8* deletion mutants (Figure 1d), confirming that the mutation in *npr‐8* is indeed responsible for the extended longevity phenotype at warm temperature. To investigate whether this phenotype is allele‐specific, we performed lifespan assays using another *npr‐8* null strain (*npr‐8(ok1446)* animals) that contains a deletion mutation in the *npr‐8* gene at a different location than the one in *npr‐8(ok1439)* animals (Consortium, 2012). Our results showed that, like *npr‐8(ok1439)* animals, *npr‐8(ok1446)* animals also lived longer than wild‐type animals at 25°C but had wild‐type lifespan at 20 and 15°C (Figure S1), indicating that the extended lifespan phenotype is not allele‐specific but is caused by the general lack of NPR‐8 function. These results suggest that NPR‐8 suppresses lifespan at warm temperature, contributing to the inhibitory effect of warm temperature on longevity.

We next examined whether NPR‐8 is required for heat stress tolerance at 35°C. To this end, synchronized wild‐type and *npr‐8(ok1439)* worms were grown to adulthood at 20°C. One‐day‐old adult worms were then transferred to 35°C and scored for survival over time. Our result showed that *npr‐8(ok1439)* worms survived significantly longer than wild‐type worms (Figure S2 and Table S1), indicating that functional loss of NPR‐8 promotes heat stress tolerance. This is consistent with the improved‐survival phenotype of *npr‐8* mutants under other stress conditions such as the warm temperature 25°C or pathogen infection (Sellegounder et al., 2019).

### 2.2 The NPR‐8‐dependent longevity response to warm temperature is neurally regulated

By using the transcriptional reporter strain *npr‐8p::gfp*, we have previously shown that NPR‐8 is expressed in the amphid sensory neurons AWB, ASJ, and AWC, as well as possibly in the lower gut region (Sellegounder et al., 2019). We were not able to rule out the possibility of the gut fluorescence being background fluorescence conferred by the backbone of the plasmid *pPD95.77* (Sellegounder et al., 2019). To determine whether the expression pattern of NPR‐8 changes with temperature, we performed fluorescent imaging of *npr‐8p::gfp* worms propagated at 20 and 25°C. Our result showed that under both temperatures, NPR‐8 was expressed in AWB, ASJ, and AWC neurons, as well as possibly in the lower gut region (Figure S3), suggesting that the NPR‐8 expression pattern did not change under these temperatures.

**FIGURE 2:**
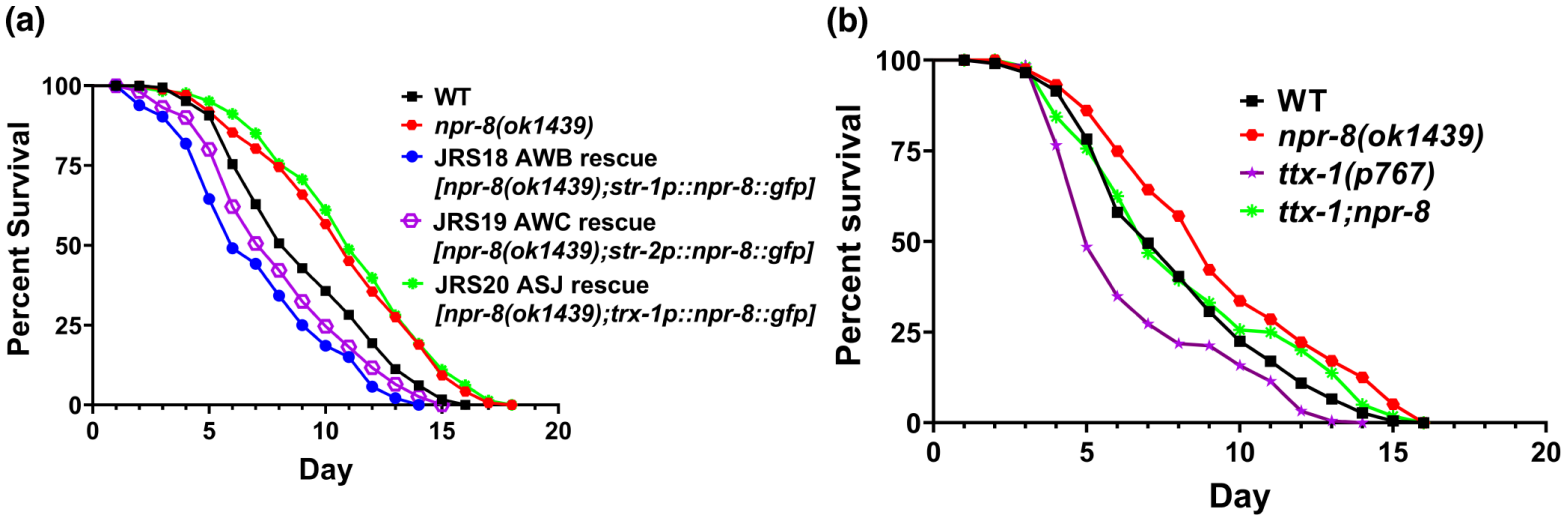
NPR‐8‐dependent longevity response to warm temperature is neurally regulated. (a) WT, *npr‐8 (ok1439)*, and rescue animals were grown at 25°C and scored for survival over time. JRS18, *npr‐8* expression rescued in AWB neurons (genotype: *npr‐8(ok1439);str‐1p::npr‐8::gfp*); JRS19, *npr‐8* expression rescued in AWC neurons (genotype: *npr‐8(ok1439);str‐2p::npr‐8::gfp*); and JRS20, *npr‐8* expression rescued in ASJ neurons (genotype: *npr‐8(ok1439);trx‐1p::npr‐8::gfp*). The graphs are the combined results of three independent experiments. Each experiment included *n* = 60 adult animals per strain. *p*‐values are relative to *npr‐8 (ok1439)*: WT, *p* < 0.0001; JRS18, *p* < 0.0001; JRS19, *p* < 0.0001; JRS20, *p* = 0.3476. (b) WT, *npr‐8(ok1439)*, *ttx‐1(p767)*, *ttx‐1;npr‐8* animals were grown at 25°C and scored for survival over time. The graphs are the combined results of three independent experiments. Each experiment included *n* = 60 adult animals per strain. *p*‐values relative to *npr‐8(ok1439)*: WT, *p* = 0.0001; *ttx‐1(p767)*, *p* < 0.0001; *ttx‐1;npr‐8*, *p* = 0.0101. *p*‐values relative to WT: *ttx‐1(p767)*, *p* < 0.0001; *ttx‐1;npr‐8*, *p* = 0.1872.

Our previous study demonstrated that NPR‐8 functions in the amphid sensory neurons to regulate *C. elegans* defense against pathogen infection (Sellegounder et al., 2019). To determine whether the NPR‐8‐dependent longevity response to warm temperature is also neurally controlled, we took advantage of our established rescue strains that specifically express NPR‐8 in the individual neurons under cell‐specific promoters in *npr‐8(ok1439)* animals (Sellegounder et al., 2019). Lifespan assays at 25°C with these animals showed that re‐expressing NPR‐8 in AWB or AWC neurons (strains JRS18 and JRS19, respectively) rescued the extended lifespan phenotype of the mutants, while re‐expression of NPR‐8 in ASJ neurons (strain JRS20) had no effect on the mutants' lifespan (Figure 2a). Like *npr‐8(ok1439)* animals, all rescue animals exhibited lifespan similar to that of wild‐type animals at 20°C (except the ASJ rescue animals, which displayed slightly longer lifespan than wild type; Figure S4). These results indicate that the lack of functional NPR‐8 in AWB and AWC neurons is responsible for the mutants' extended lifespan at 25°C. In other words, NPR‐8 functions in AWB and AWC neurons to regulate the longevity response to warm temperature. It is worth noting that JRS18 AWB rescue animals and JRS19 AWC rescue animals had shorter lifespan than wild‐type animals, while JRS17 native rescue animals had lifespan comparable to wild‐type animals (Figures 1d and 2a). A plausible explanation for this phenomenon is that the levels of rescued *npr‐8* expression could be higher in JRS18 and JRS19 than that in JRS17, which may inhibit the lifespan in the former two strains. To test this possibility, we performed qRT‐PCR to measure *npr‐8* mRNA levels in these rescue strains as well as in wild‐type animals. Our results showed that compared to wild‐type animals, JRS17, JRS18, and JRS19 expressed 54‐, 226‐, and 273‐fold *npr‐8* mRNA, respectively (Figure S5). On the one hand, re‐expression of *npr‐8* in these JRS strains rescued the extended longevity phenotype of *npr‐8(ok1439)* worms, supporting our conclusion that NPR‐8 functions in AWB and AWC neurons to suppress lifespan at warm temperature. The observation that higher levels of *npr‐8* expression in JRS18 and JRS19 resulted in shorter‐than‐wild‐type lifespan in these worms is consistent with this conclusion. On the other hand, JRS17 had higher *npr‐8* expression than wild‐type animals (Figure S5) despite their comparable lifespan (Figure 1d), suggesting that there could be a threshold of *npr‐8* overexpression in connection with the lifespan phenotype.

As mentioned above, our previous work also showed that NPR‐8 is possibly expressed in the intestine in addition to its expression in the amphid sensory neurons (Sellegounder et al., 2019). To determine whether the lack of NPR‐8 expression in the intestine contributes to the lifespan phenotype of *npr‐8(ok1439)* animals, we used tissue‐specific RNAi to inhibit *npr‐8* expression, if any, in the intestine and then examined the lifespan of these animals at 25°C. To this end, we used VP303 animals that are capable of intestine‐specific RNAi for the experiments (Espelt et al., 2005). As a positive control, we also performed RNAi of *npr‐8* in neurons by using the strain TU3401 that is capable of neuron‐specific RNAi (Calixto et al., 2010). Our results showed that compared to RNAi with an empty vector, RNAi of *npr‐8* in VP303 animals had no effect on their lifespan, whereas RNAi of *npr‐8* in TU3401 animals significantly extended lifespan as expected (Figure S6). These data suggest that the lack of NPR‐8 expression in the intestine does not contribute to the temperature‐induced longevity phenotype of *npr‐8* mutant animals.

In *C. elegans*, AFD thermosensory neurons are required for maintaining lifespan at warm temperature (25°C), and ablation or inactivation of these neurons shortens the worm's lifespan (Chen et al., 2016; Lee & Kenyon, 2009). To test whether AFD neurons also function in the NPR‐8‐dependent longevity response to warm temperature, we examined how a loss‐of‐function mutation in *ttx‐1*, a gene critical for AFD differentiation and function (Satterlee et al., 2001), affected the lifespan of *npr‐8(ok1439)* animals at warm temperature. To this end, we crossed *npr‐8(ok1439)* and *ttx‐1(p767)* animals to generate *ttx‐1;npr‐8* double mutant animals, then subjected the single and double mutant animals, along with wild‐type controls, to lifespan assays at 25°C. Consistent with previous reports (Chen et al., 2016; Lee & Kenyon, 2009), the lives of thermosensory‐defective *ttx‐1(p767)* animals were shorter than those of wild‐type animals (Figure 2b). Interestingly, *ttx‐1;npr‐8* double mutant animals displayed longer lifespan than *ttx‐1(p767)* animals but shorter lifespan than *npr‐8(ok1439)* animals (Figure 2b), indicating that the *ttx‐1* and *npr‐8* mutations reduce each other's effect on longevity at warm temperature. These results suggest that the warm temperature‐induced longevity response is regulated by a circuit of thermosensory and chemosensory neurons working in an antagonistic manner.

Additionally, we also tested if AFD neurons affect the lifespan of *npr‐8(ok1439)* animals at 15°C and 20°C using wild‐type, *npr‐8(ok1439)*, *ttx‐1(p767)*, and *ttx‐1(p767);npr‐8(ok1439)* animals. Consistent with what we have already observed (Figure 1b,c), wild‐type and *npr‐8(ok1439)* animals had similar lifespan at 15°C or 20°C (Figure S7). Interestingly, *ttx‐1(p767)* and *ttx‐1(p767);npr‐8(ok1439)* animals also displayed wild‐type lifespan at 15°C or 20°C, indicating that functional loss of NPR‐8 and/or AFD neurons had no effects on longevity under these temperatures (Figure S7). This result is consistent with an independent study showing that the AFD thermosensory circuit regulates longevity at 25°C but not at 15°C or 20°C (Lee & Kenyon, 2009).

### 2.3 Both warm temperature and old age broadly alter gene expression in *C. elegans*

To gain molecular insights into the NPR‐8‐dependent longevity response to temperature, we employed RNA‐seq to compare gene expression in young and old wild‐type and *npr‐8(ok1439)* animals propagated at different temperatures. To this end, we collected five replicates of eight groups of RNA samples (1‐day‐old and 9‐day‐old wild‐type and *npr‐8(ok1439)* adults propagated at 20°C and 25°C; Table 1). These samples were then submitted to the Washington State University Genomics Core for RNA‐seq analysis. A summary of our RNA‐seq analysis is listed in Table 2.

**TABLE 1:** Grouping of RNA‐seq samples.

Sample group	Strain	Propagation temperature (°C)	Adult age
1	*N2*	20	1‐day‐old
2	*N2*	20	9‐day‐old
3	*N2*	25	1‐day‐old
4	*N2*	25	9‐day‐old
5	*npr‐8(ok1439)*	20	1‐day‐old
6	*npr‐8(ok1439)*	20	9‐day‐old
7	*npr‐8(ok1439)*	25	1‐day‐old
8	*npr‐8(ok1439)*	25	9‐day‐old

**TABLE 2:** Summary of RNA‐seq analysis.

Influencing factor	Comparison	Sample group	Number of regulated genes	Gene ontology (GO) analysis		Data location
Effects of temperature on gene expression	1‐day‐old wild‐type animals at 25°C relative to 20°C	Group 3 vs. Group 1	2265 genes upregulated	340 enriched biological processes	Cell cycle, reproduction, metabolic/biosynthetic processes, organelle organization, development, DNA replication/repair/transcription/recombination, and other processes	Table S2 Table S8, ^a^
			5069 genes downregulated	68 attenuated biological processes	Signaling, phosphorylation/dephosphorylation, immune response, ion/transmembrane transport, and other processes	Table S3 Table S4 Table S9, ^a^ Table S14, ^a^
Effects of aging on gene expression	Wild‐type animals at 20°C day 9 relative to day 1	Group 2 vs. Group 1	3792 genes upregulated	428 enriched biological processes	Cell cycle, reproduction/development, metabolism, organelle organization, DNA replication/repair/transcription, and other processes	Table S5 Table S10, ^a^
			4937 genes downregulated	56 attenuated biological processes	Ion/transmembrane transport, phosphorylation/dephosphorylation, signaling, immune response, and other processes	Table S6 Table S11, ^a^
				65 attenuated molecular functions	“Structural constituent of cuticle” is the most significantly reduced	Table S7 Table S12, ^a^
Effects of *npr‐8* mutation on gene expression	1‐day‐old *npr‐8(ok1439)* animals at 20°C relative to wild‐type controls	Group 5 vs. Group 1	643 genes upregulated	7 enriched molecular functions	“Structural constituent of cuticle” is the most significantly enriched	Table S15
			272 genes downregulated	No attenuated GO terms		
	9‐day‐old *npr‐8(ok1439)* animals at 20°C relative to wild‐type controls	Group 6 vs. Group 2	1487 genes upregulated	33 enriched biological processes	Phosphorylation/dephosphorylation, metabolic process	Table S16
			1305 genes downregulated	13 attenuated molecular functions	“Structural constituent of cuticle” is the most significantly reduced	Table 3
	1‐day‐old *npr‐8(ok1439)* animals at 25°C relative to wild‐type controls	Group 7 vs. Group 3	1482 genes upregulated	17 enriched molecular functions	“Structural constituent of cuticle” is the most significantly enriched	Table S17
			419 genes downregulated	No attenuated GO terms		
	9‐day‐old *npr‐8(ok1439)* animals at 25°C relative to wild‐type controls	Group 8 vs. Group 4	1307 genes upregulated	29 enriched biological processes	Phosphorylation/dephosphorylation, metabolic process	Table S18
			631 genes downregulated	No attenuated GO terms		

a Tables were generated based on RNA‐seq analysis with 1% FDR. All other tables and all other data in Table 2 were generated based on RNA‐seq analysis with 5% FDR.

Using the RNA‐seq data, we first assessed how propagation temperature affects gene expression by comparing the expression profiles of 1‐day‐old wild‐type adults grown at 25°C with those at 20°C (Group 3 vs. Group 1 in Table 1). In total, 16,135 genes were identified and quantified with a false discovery rate (FDR) of 5%. Among these genes, 2265 were upregulated and 5069 were downregulated at least twofold in animals grown at 25°C relative to those grown at 20°C. The changes in expression of almost half of the total identified genes (45%) indicate that growth temperature profoundly affects gene expression in *C. elegans*. This might explain, at the molecular level, why temperature is so crucial for life in that even modest variations in temperature can alter many aspects of behavior and physiology including aging (Keil et al., 2015). Gene ontology (GO) analysis of the 2265 upregulated genes identified 340 significantly enriched biological processes, which can be loosely divided into seven categories: cell cycle, reproduction, metabolic/biosynthetic processes, organelle organization, development, DNA replication/repair/transcription/recombination, and other processes (Table S2). GO analysis of the 5069 downregulated genes identified 68 significantly attenuated biological processes, which can be loosely divided into five categories: signaling, phosphorylation/dephosphorylation, immune response, ion/transmembrane transport, and other processes (Table S3). It is not clear which regulated biological processes are responsible for the shortened lifespan of *C. elegans* at warm temperature. The enrichment of genes involved in metabolic/biosynthetic processes may reflect an increased rate of metabolism at 25°C relative to 20°C (Table S2), which could provide a molecular basis for the rate of living theory. It is worth noting that among the downregulated biological processes (Table S3), signaling is the most significantly reduced and includes reduction in GPCR signaling pathway (GO term GO:0007186), neuropeptide signaling pathway (GO:0007218), synaptic signaling (GO:0099536), nervous system process (GO:0050877), and other signaling processes. A total of 523 genes encoding signaling molecules such as GPCRs and neuropeptides as well as enzymes involved in the metabolism of neurotransmitters or neurohormones contribute to the downregulation of signaling processes (Table S4). These data demonstrate that signaling processes, especially those controlled by the nervous system, are reduced in animals propagated at 25°C compared to those propagated at 20°C, indicating a potential connection between neural signaling and longevity in *C. elegans*. Indeed, signaling from AFD thermosensory neurons and their downstream AIY interneurons are required for maintaining lifespan at 25°C (Chen et al., 2016; Lee & Kenyon, 2009). However, detailed information about neural circuits that control the longevity response to temperature is lacking.

We next examined how aging affects gene expression by comparing the expression profiles of 9‐day‐old wild‐type adult animals with those of 1‐day‐old adults grown at 20°C (Group 2 vs. Group 1 in Table 1). We found that 3792 genes were upregulated, and 4937 genes were downregulated at least twofold in old animals relative to young adults. The changes in expression of more than half of the total identified genes indicate that, like temperature, age also has profound effects on *C. elegans* behavior and physiology. GO analysis of the 3792 upregulated genes identified 428 significantly enriched biological processes, which can be loosely divided into six categories: cell cycle, reproduction/development, metabolism, organelle organization, DNA replication/repair/transcription, and other processes (Table S5). GO analysis of the 4937 downregulated genes identified 56 significantly attenuated biological processes, which can be loosely divided into five categories: ion/transmembrane transport, phosphorylation/dephosphorylation, signaling, immune response, and other processes (Table S6). These data show that similar biological processes are regulated by old age and warm temperature, suggesting that both conditions could be stressful to *C. elegans*. Interestingly and importantly, GO analysis of the downregulated genes also revealed 65 attenuated molecular functions, with “structural constituent of cuticle” being the most significantly reduced (Table S7A). One hundred and seven downregulated genes contribute to the reduction in cuticle structure activity, among which 88 encode cuticular collagens (Table S7B), indicating that cuticular collagens could be essential for aging and longevity. Indeed, previous studies have shown that the expression levels of collagen genes decline with age and that increased collagen expression is a common feature of multiple conserved longevity pathways and essentially every longevity intervention (Budovskaya et al., 2008; Ewald et al., 2015). Cuticular collagens have also been implicated in lengthening *C. elegans* lifespan under stress such as pathogen infection and oxidative stress (Ewald et al., 2015; Sellegounder et al., 2019).

The above‐described RNA‐seq analyses were done with an FDR of 5%. We also performed similar analyses with a more stringent FDR of 1%. As expected, the numbers of upregulated and downregulated genes decreased. GO analysis of these upregulated and downregulated genes revealed essentially the same enriched and attenuated biological processes, respectively, as in the analysis with 5% FDR (for the temperature effects, compare Table S2 vs. Table S8 and Table S3 vs. Table S9; for the age effects, compare Table S5 vs. Table S10 and Table S6 vs. Table S11). Importantly, GO analysis of the downregulated genes in the dataset for assessing the age effects (9‐day‐old vs. 1‐day‐old wild‐type adults grown at 20°C) also revealed 65 attenuated molecular functions, with “structural constituent of cuticle” being the most significantly reduced (Table S12). One hundred collagen genes were downregulated and contributed to the attenuation of cuticle structure activity (Table S12). These results recapitulated the age effects from the analysis with 5% FDR (Table S7). Taken together, both analyses with 1% and 5% FDRs revealed the same important gene regulations exerted by temperature or age.

To identify the most highly and significantly changed genes, we generated two volcano plots: one plot from the dataset for assessing the age effects (9‐day‐old vs. 1‐day‐old wild‐type adults grown at 20°C) and the other from the dataset for assessing the temperature effects (1‐day‐old wild‐type animals grown at 20°C vs. 25°C) (Figure S8). We arbitrarily set *p*‐value cutoff at 10e‐100 and log2FoldChange cutoff at 5 on the volcano plots (Figure S8). From the dataset for assessing the age effects, we obtained 126 and 687 genes that were upregulated and downregulated, respectively (Figure S8A and Table S13). While GO analysis of the 126 upregulated genes did not yield significantly enriched GO terms, a similar analysis of the 687 downregulated genes revealed “structural constituent of cuticle” being the most significantly attenuated molecular function, with 62 collagen genes contributing to such attenuation (Table S13). These collagen genes were among those contributing to the attenuation of the same molecular function in the RNA‐seq analysis with 1% FDR (compare Table S12 vs. Table S13), indicating that the changes in collagen gene expression are important and that the volcano plot recapitulated these changes in the RNA‐seq data.

Similarly, from the volcano plot for assessing the temperature effects, we obtained 85 and 234 genes that were upregulated and downregulated, respectively (Figure S8B and Table S13). GO analysis of the 85 upregulated genes revealed only one enriched biological process, namely P granule organization, with four genes contributing to such enrichment (Table S13). P granules are germ granules in *C. elegans* that contain a heterogeneous mixture of RNAs and proteins associated with germ line proliferation and gametogenesis (Updike & Strome, 2010). Given the inherent sensitivity of the germ line to temperature, it is not surprising that P granule components changed dramatically under the different propagation temperatures (Spike et al., 2008). Despite its great change determined by the volcano plot, P granule organization was not an enriched GO term in our RNA‐seq analysis with 1% FDR (Table S8), possibly due to the unranked differentially expressed gene (DEG) lists being used in that analysis, whereas DEGs were ranked based on fold change and significance in the volcano plot analysis (Eden et al., 2009). Nonetheless, this important finding warrants further investigation. GO analysis of the 234 downregulated genes revealed the GO term “structural constituent of cuticle” being the most significantly attenuated molecular function, with 61 collagen genes contributing to the attenuation (Table S13). Such reduction in collagen gene expression under warm temperature was also discovered in our RNA‐seq analysis with 1% FDR, although “structural constituent of cuticle” was not the most significantly attenuated GO term or molecular function in this analysis (Table S14), likely due to the unranked DEG lists being used in the analyses (Eden et al., 2009). Taken together, the volcano plots highlighted the changes of collagen expression being important gene regulation impacted by both temperature and age.

To determine how gene expression differed among the samples and which factor had the greatest effect on gene expression, we performed principal component analysis (PCA) of our RNA‐seq data from all 40 samples (five replicates of eight groups of samples, i.e., young and old wild‐type and *npr‐8(ok1439)* animals propagated at 20 and 25°C, Table 1). Our PCA result showed that young animals were separated from old animals by age in the first component (88% variance), and that the young animals were further separated among themselves in the second component based on propagation temperatures (7% variance) (Figure S9A). *npr‐8(ok1439)* animals were separated from wild‐type animals in the first component only at young age at 25°C, possibly reflecting the mutants' younger‐than‐wild‐type biological age (Figure S9A). At the old age, however, all 20 samples of old age were clustered together regardless of propagation temperature or mutation (Figure S9A). These results suggest that among all the factors influencing gene expression, the old age had the greatest effect and was a converging point of various impacts on gene expression. Of note, replicate #2 in sample group 1 (young wild‐type worms propagated at 20°C) appeared to distance itself from the group, and all other samples were closely clustered within their own groups, indicating overall good consistency and similarity among the sample replicates (Figure S9A). To determine whether replicate #2 in group 1 was a true outlier, we calculated the mean of read counts of all the genes in each of the five replicates in group 1 and then performed *t*‐tests to compare the replicates following our established method of assessing concordance between replicate samples (Wang et al., 2022). As shown in Figure S9B, the gene read counts were comparable among the five replicates, and the *t*‐tests did not find a significant difference between any two replicates. These data indicate that replicate #2 in group 1 was not an outlier and that it should be included in data analysis as we did in our study.

### 2.4 NPR‐8 differentially regulates collagen genes in an age‐ and temperature‐dependent manner

To assess the role of NPR‐8 in the temperature‐induced longevity response, we compared the expression profiles of young and old *npr‐8(ok1439)* animals with those of wild‐type animals maintained at the different propagation temperatures. For 1‐day‐old adults grown at 20°C, 643 genes were upregulated and 272 genes were downregulated at least twofold in *npr‐8(ok1439)* animals relative to wild‐type controls. While GO analysis of the 272 downregulated genes did not yield significantly attenuated biological processes or molecular functions, a similar analysis of the 643 upregulated genes identified seven significantly enriched molecular functions, with the GO term “structural constituent of cuticle” being the most significantly enriched (Table S15A). Sixteen upregulated genes were related to cuticle structure activity, nine of which encoded cuticular collagens (Table S15B). These data indicate that at the normal growth temperature of 20°C, NPR‐8 suppresses the expression of a subset of cuticular collagen genes in young wild‐type animals. This result is consistent with our previous study showing that NPR‐8 suppresses the basal expression of collagen genes (Sellegounder et al., 2019). For 9‐day‐old adult animals grown at 20°C, 1487 genes were upregulated and 1305 genes were downregulated at least twofold in *npr‐8(ok1439)* animals relative to wild‐type controls. The total number of regulated genes tripled at old age compared to young age, indicating that the effects of NPR‐8 on *C. elegans* physiology are amplified by aging. GO analysis of the 1487 upregulated genes identified 33 significantly enriched biological processes, which can be divided into three categories: phosphorylation/dephosphorylation, metabolic process, and other processes (Table S16). GO analysis of the 1305 downregulated genes revealed 13 attenuated molecular functions, with the GO term “structural constituent of cuticle” being the most significantly reduced (Table 3a). Seventy‐three downregulated genes contributed to the decrease in cuticle structure activity, 60 of which encoded cuticular collagens (Table 3b). These data indicate that at old age, NPR‐8 upregulates the expression of cuticular collagens in wild‐type animals, which is surprising and is in direct contrast with NPR‐8's function at young age when it suppresses cuticular collagens (Table S15). More surprisingly, eight of the nine collagen genes (except *col‐98*) that changed expression at young age were also regulated at old age (compare Table 3b to Table S15B). These results are unexpected and raised a perplexing question, that is, how does NPR‐8, a single type of GPCR, exerts opposite effects on collagen expression at different stages of the *C. elegans* life cycle? Does *npr‐8* influence both fitness and aging similar to genes described in George Williams' antagonistic pleiotropic theory that have beneficial effects on fitness in early life but are detrimental in later life (Williams, 1957)?

**TABLE 3:** GO analysis of downregulated genes in 9‐day‐old *npr‐8(ok1439)* animals grown at 20°C relative to wild‐type animals.

(A) Attenuated molecular functions				
GO term	Description	*p*‐Value^a^	FDR q‐value^b^	Enrichment (*N*, *B*, *n*, *b*)^c^
GO:0042302	Structural constituent of cuticle	4.74E‐49	1.22E‐45	7.67 (10804, 131, 785, 73)
GO:0005198	Structural molecule activity	9.30E‐22	1.20E‐18	3.19 (10804, 349, 785, 81)
GO:0008239	Dipeptidyl‐peptidase activity	5.97E‐06	5.12E‐03	8.03 (10804, 12, 785, 7)
GO:0005201	Extracellular matrix structural constituent	3.81E‐05	2.45E‐02	4.95 (10804, 25, 785, 9)
GO:0000978	RNA polymerase II proximal promoter sequence‐specific DNA binding	5.50E‐05	2.83E‐02	1.94 (10804, 269, 785, 38)
GO:0030246	Carbohydrate binding	6.65E‐05	2.85E‐02	2.23 (10804, 167, 785, 27)
GO:0000987	Proximal promoter sequence‐specific DNA binding	7.01E‐05	2.57E‐02	1.92 (10804, 272, 785, 38)
GO:0000977	RNA polymerase II regulatory region sequence‐specific DNA binding	7.47E‐05	2.40E‐02	1.82 (10804, 333, 785, 44)
GO:0001012	RNA polymerase II regulatory region DNA binding	7.47E‐05	2.13E‐02	1.82 (10804, 333, 785, 44)
GO:0016634	Oxidoreductase activity, acting on the CH‐CH group of donors, oxygen as acceptor	9.31E‐05	2.39E‐02	8.60 (10804, 8785, 5)
GO:0016491	Oxidoreductase activity	1.45E‐04	3.39E‐02	1.62 (10804, 502, 785, 59)
GO:0097602	Cullin family protein binding	1.93E‐04	4.14E‐02	4.59 (10804, 24, 785, 8)
GO:0000981	DNA‐binding transcription factor activity, RNA polymerase II‐specific	2.04E‐04	4.03E‐02	1.77 (10804, 327, 785, 42)

(B) Downregulated genes related to the reduced cuticle structure activity					
Genes	Fold change	Adjusted *p*‐value^d^	Genes	Fold change	Adjusted *p*‐value^d^
rol‐1	15.3	1.73E‐02	col‐125	4.7	1.61E‐04
col‐139	9.3	1.61E‐04	col‐101	4.5	1.61E‐04
col‐73	9.2	1.61E‐04	col‐144	4.2	1.61E‐04
col‐146	8.9	3.24E‐02	col‐169	4.2	1.61E‐04
col‐77	8.3	1.61E‐04	col‐41	4.2	5.99E‐04
col‐179	8.1	1.61E‐04	sqt‐3	4	1.61E‐04
col‐49	7.9	1.61E‐04	col‐103	3.9	1.61E‐04
dpy‐13	7.7	2.89E‐02	col‐10	3.8	1.61E‐04
col‐129	7.6	1.61E‐04	col‐122	3.7	1.61E‐04
col‐156	7.5	2.86E‐02	sqt‐2	3.7	1.61E‐04
col‐20	7.5	1.61E‐04	col‐161	3.7	1.61E‐04
bli‐6	7.4	1.61E‐04	col‐181	3.6	1.61E‐04
col‐138	7.4	1.61E‐04	col‐143	3.6	1.61E‐04
col‐124	7.3	1.61E‐04	col‐184	3.6	1.61E‐04
col‐90	7.2	1.61E‐04	Y69H2.14	3.5	1.61E‐04
dpy‐4	7.2	1.61E‐04	col‐166	3.4	1.61E‐04
col‐147	7.2	1.61E‐04	col‐110	3.4	1.08E‐02
col‐140	7.2	1.61E‐04	cut‐2	3.4	1.61E‐04
col‐168	7.1	1.61E‐04	col‐145	3	1.61E‐04
dpy‐5	7	1.61E‐04	col‐12	3	1.61E‐04
col‐119	7	1.61E‐04	col‐155	2.9	1.61E‐04
col‐159	6.9	1.61E‐04	col‐88	2.8	1.61E‐04
col‐80	6.7	1.61E‐04	col‐14	2.8	1.61E‐04
col‐160	6.6	1.61E‐04	col‐99	2.8	1.61E‐04
col‐178	6.4	1.61E‐04	col‐54	2.6	1.80E‐02
rol‐6	6.3	1.61E‐04	col‐65	2.5	5.99E‐04
rol‐8	6.3	1.61E‐04	col‐153	2.4	1.61E‐04
col‐142	6.3	1.61E‐04	let‐653	2.4	1.61E‐04
col‐92	6.2	4.70E‐02	col‐118	2.2	1.61E‐04
col‐94	6.2	4.42E‐02	dpy‐8	2.2	1.61E‐04
col‐19	5.8	1.61E‐04	col‐173	2.1	4.38E‐03
col‐133	5.7	1.61E‐04	col‐176	2	5.44E‐03
col‐149	5.5	1.61E‐04	col‐157	2	3.12E‐04
col‐167	5.2	1.61E‐04	col‐62	2	8.26E‐03
col‐170	5.2	9.91E‐03	col‐130	2	3.07E‐02
col‐13	5.1	1.02E‐02	col‐150	2	2.18E‐03
col‐180	4.8	1.61E‐04			

^a^ *P*‐Value is computed according to the mHG model.

^b^ FDR *q*‐value is the correction of the above *p*‐value for multiple testing using the Benjamini and Hochberg method.

^c^ Enrichment (*N*, *B*, *n*, *b*) is defined as follows: *N*—total number of genes; *B*—total number of genes associated with a specific GO term; *n*—number of genes in the target set; *b*—number of genes in the intersection;Enrichment = (*b*/*n*)/(*B*/*N*).

^d^ Adjusted *p* value is the correction of the *p* value for multiple testing using the Benjamini and Hochberg method.

We next examined the gene expression profiles of young and old *npr‐8(ok1439)* and wild‐type animals grown at 25°C. For 1‐day‐old adults, 1482 genes were upregulated and 419 genes were downregulated at least twofold in *npr‐8(ok1439)* animals relative to wild‐type animals. GO analysis of the 1482 upregulated genes identified 17 enriched molecular functions, with the GO term “structural constituent of cuticle” being the most significantly enriched (Table S17A). Seventy upregulated genes were related to cuticle structure activity, 56 of which encoded cuticular collagens (Table S17B). These data indicate that, similar to 1‐day‐old adults grown at 20°C (Table S15), NPR‐8 also suppresses the expression of cuticular collagen genes in young adults grown at 25°C. However, there are many more collagen genes suppressed at 25°C than at 20°C (56 genes vs. 9 genes, compare Table S17B with Table S15B), indicating that warm temperature broadens NPR‐8's inhibitory effect on collagen gene expression in young animals. For 9‐day‐old adults, 1307 genes were upregulated and 631 genes were downregulated at least twofold in *npr‐8(ok1439)* animals relative to wild‐type animals. GO analysis of the 1307 upregulated genes revealed 29 significantly enriched biological processes, which can be divided into three categories: phosphorylation/dephosphorylation, metabolic process, and other processes (Table S18). These data demonstrate that in old animals, the biological processes suppressed by NPR‐8 at 25°C are similar to those suppressed by NPR‐8 at 20°C (compare Table S18 with Table S16), indicating that these specific NPR‐8 effects are age‐related, not temperature‐dependent. GO analysis of the 631 downregulated genes did not yield any significantly enriched biological processes or molecular functions. This is in striking contrast with old worms grown at 20°C in which a large number of cuticular collagen genes (60 genes) were downregulated in *npr‐8(ok1439)* animals relative to wild‐type animals (Table 3b). Because collagen expression promotes longevity (Budovskaya et al., 2008; Ewald et al., 2015), this lack of downregulation of collagen expression at 25°C may explain the extended lifespan of *npr‐8(ok1439)* animals at warm temperature.

### 2.5 NPR‐8‐regulated collagen genes contribute to the extended lifespan of *npr‐8(ok1439)* animals at 25°C

**FIGURE 3:**
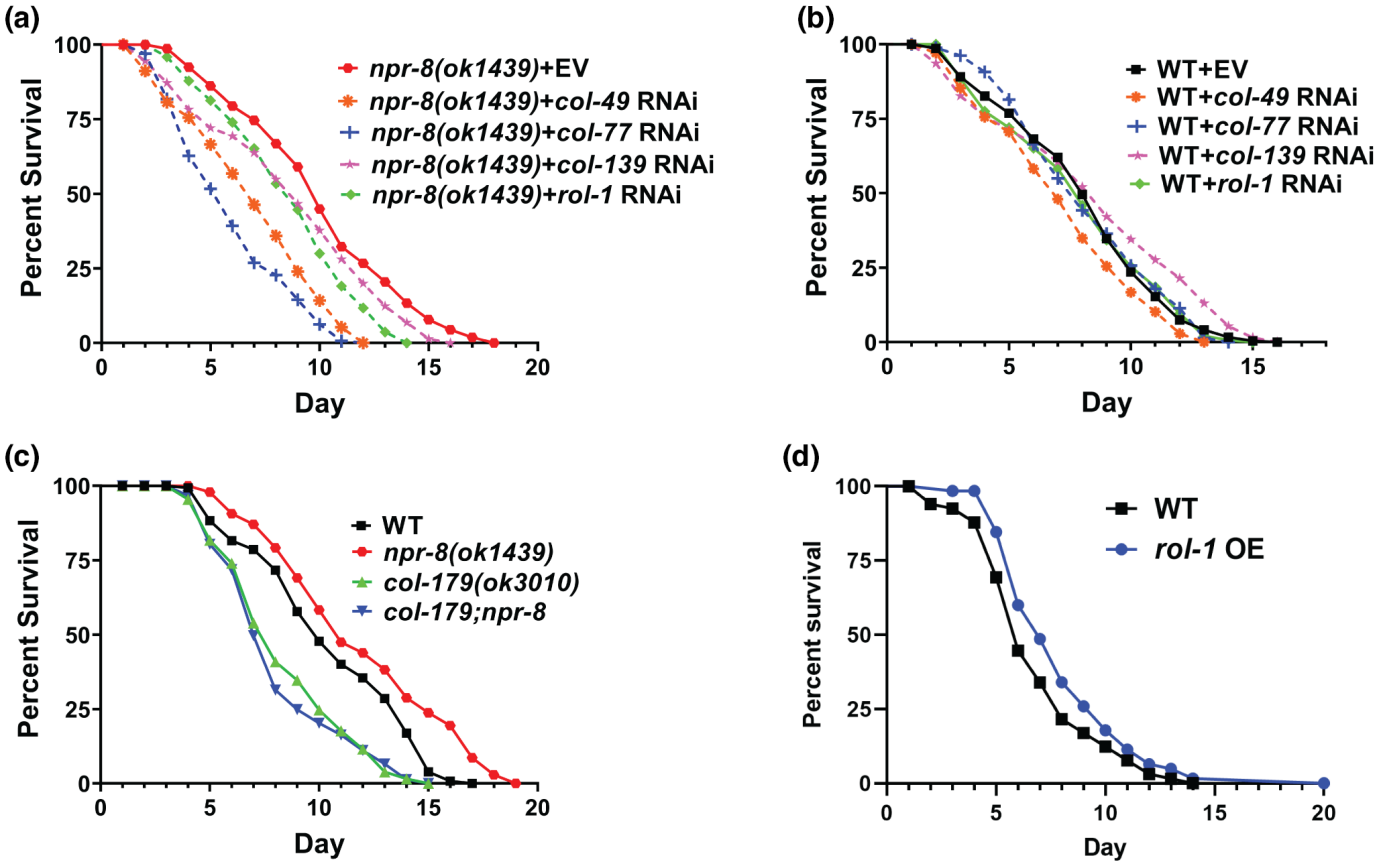
NPR‐8‐regulated collagen genes are required for warm temperature‐induced lifespan extension in *npr‐8(ok1439)* animals. *npr‐8(ok1439)* animals (a) and WT animals (b) were grown on dsRNA for empty vector (EV) or for *rol‐1*, *col‐49*, *col‐77*, or *col‐139* at 25°C and scored for survival over time. The graphs are the combined results of three independent experiments. Each experiment included *n* = 60 adult animals per strain. *p*‐values are relative to *npr‐8(ok1439)* + EV in (a): *npr‐8(ok1439)* + *col‐49*, *p* < 0.0001; *npr‐8(ok1439)* + *col‐77*, *p* < 0.0001; *npr‐8(ok1439)* + *col‐139*, *p* = 0.0009; *npr‐8 (ok1439)* + *rol‐1*, *p* < 0.0001. *p*‐values are relative to WT + EV in (b): WT + *col‐49*, *p* = 0.0039; WT + *col‐77*, *p* = 0.9225; WT + *col‐139*, *p* = 0.0261; WT + *rol‐1*, *p* = 0.8383. (c) WT, *npr‐8(ok1439)*, *col‐179(ok3010)*, *npr‐8;col‐179* animals were grown at 25°C and scored for survival over time. The graphs are the combined results of three independent experiments. Each experiment included *n* = 60 adult animals per strain. *p‐*values relative to *npr‐8(ok1439)*: WT, *p* < 0.0001; *col‐179(ok3010)*, *p* < 0.0001; *npr‐8;col‐179*, *p* < 0.0001. *p‐*values relative to WT: *col‐179(ok3010)*, *p* < 0.0001; *npr‐8;col‐179*, *p* < 0.0001. (d) WT and *rol‐1* overexpressing strain LYY13 (genotype: *rol‐1p::rol‐1gDNA::gfp*) were grown at 25°C and scored for survival over time. The graphs are the combined results of three independent experiments. Each experiment included *n* = 60 adult animals per strain. The *p*‐value between the two strains is 0.0453.

To determine whether collagen expression plays a role in *npr‐8(ok1439)* animals' extended lifespan at warm temperature, we inactivated NPR‐8‐regulated collagen genes using RNA interference (RNAi) or deletion mutation in *npr‐8(ok1439)* and wild‐type animals and then performed lifespan assays at 25°C on these animals. For the five most‐regulated collagen genes tested (downregulated most in *npr‐8(ok1439)* animals at 20°C but lacking such downregulation at 25°C, Table 3b), inactivation of each of them (*rol‐1*, *col‐49*, *co‐77*, *col‐139*, or *col‐179*) significantly suppressed the extended lifespan of *npr‐8* mutant animals at 25°C (Figure 3a,c). Inactivation of *col‐49* and *col‐179* also suppressed the lifespan of wild‐type animals (Figure 3b). These results indicate that the expression of NPR‐8‐regulated collagen genes contributes to the extended lifespan of *npr‐8(ok1439)* animals at 25°C and is also essential for maintaining the lifespan of wild‐type animals at warm temperature. This is consistent with the longevity‐promoting role of collagens in the aging process (Budovskaya et al., 2008; Ewald et al., 2015).

To further investigate the roles of NPR‐8‐regulated collagens in longevity, we attempted to generate two transgenic worm strains that overexpress *rol‐1* and *col‐49*, two of the most highly regulated collagen genes examined in our gene inactivation experiment. These strains were constructed using our published protocol (Sellegounder et al., 2019), and stable lines were established (i.e., strain LYY13 for *rol‐1* and strain LYY14 for *col‐49*). However, compared to the wild‐type expression levels, only *rol‐1* overexpression in LYY13 was significant, whereas *col‐49* overexpression in LYY14 was not, as confirmed by qRT‐PCR (Figure S10A). Lifespan assays at 25°C showed that LYY13 worms lived significantly longer than wild‐type worms (Figure 3d) while LYY14 had the same lifespan as wild‐type worms (Figure S10B). These data indicate that overexpression of NPR‐8‐regulated collagen genes was sufficient to extend lifespan at 25°C. In contrast to the lifespan assays at 25°C, however, lifespan assays at 20°C showed no impact of overexpressing *rol‐1* on worm lifespan (Figure S10C). Taken together, these data suggest that collagen expression preferentially enhances survival at the warm temperature, consistent with its role in enhanced stress resistance to various environmental stimuli such as oxidative stress and pathogen infection (Ewald et al., 2015; Sellegounder et al., 2019).

In addition to collagen genes, the genes upregulated in *npr‐8(ok1439)* animals at 25°C but not at 20°C in Day 1 could also contribute to the mutants' extended lifespan at warm temperature. To test this possibility, we first identified such genes by comparing the upregulated genes in *npr‐8(ok1439)* animals relative to wild‐type animals at 25°C in Day 1 with those upregulated at 20°C in Day 1 and obtained 1331 genes that were only upregulated at 25°C (Table S19). GO analysis of these 1331 genes revealed that “structural constituent of cuticle” was the most significantly enriched molecular function with 65 collagen genes contributing to such enrichment (Table S20), indicating a potential connection between collagen upregulation and lifespan extension at 25°C. To determine whether the upregulated gene expression in *npr‐8(ok1439)* animals at 25°C but not at 20°C in Day 1 contributed to the mutants' lifespan extension at 25°C, we inactivated five most upregulated genes individually by RNAi in wild‐type and *npr‐8(ok1439)* animals and then assayed their lifespan at 25°C. These include genes encoding the Hedgehog‐like protein GRL‐21, the fibronectin‐like protein C42D4.3, the cuticlin CUT‐2, and the two collagens COL‐7 and COL‐88 (Table S19). Our lifespan assays showed that compared to the control RNAi with an empty vector, RNAi of each of these genes shortened the extended lifespan of *npr‐8(ok1439)* animals at 25°C and that RNAi of four genes also suppressed the lifespan of wild‐type animals, although to a lesser extent compared to that of *npr‐8* mutants (Figure S11). These results indicate that the upregulated gene expression in *npr‐8(ok1439)* animals at 25°C but not at 20°C in Day 1 contributed to the mutants' lifespan extension at 25°C, and that some of the gene expression was also essential for maintaining the lifespan of wild‐type animals at warm temperature. The lifespan data derived from collagen genes *col‐7* and *col‐8*, as well as from *cut‐2* (like collagen genes, *cut‐2* encodes a component of the cuticle), support the notion that collagen expression promotes longevity. The physiological functions of GRL‐21 and C42D4.3 are largely unknown. GRL‐21 is expressed in the hypodermis and has been implicated in pathogen resistance (Lin & Wang, 2017; Zarate‐Potes et al., 2022). C42D4.3 is a component of extracellular matrix (ECM) and is also involved in the innate immune response to pathogen infection (Burton et al., 2021; Styer et al., 2008). In short, like collagens, GRL‐21 and C42D4.3 are ECM‐related and are involved in stress resistance. Our data revealed that all these ECM‐related genes were regulated by NPR‐8 and promoted longevity at 25°C.

We also investigated which transcription factors control the gene expression in the NPR‐8‐dependent response. To this end, two common longevity‐regulatory transcription factors DAF‐16 and SKN‐1 were tested (Uno & Nishida, 2016). Our results showed that inactivating *skn‐1* through RNAi had no effects on the lifespan of *npr‐8(ok1439)* animals at 25°C, whereas a *daf‐16* deletion mutation shortened the extended lifespan of *npr‐8* mutant animals to below the wild‐type level (Figure S12), indicating that DAF‐16 is involved in the NPR‐8‐dependent longevity response to warm temperature.

To analyze the subcellular localization of DAF‐16 in *npr‐8(ok1439)* worms, we crossed *npr‐8(ok1439)* worms with the DAF‐16::GFP reporter strain TJ356 (Henderson & Johnson, 2001) to create strain LYY15 [*npr‐8(ok1439);zls356*] that expresses DAF‐16::GFP in a *npr‐8* mutant background. We then imaged DAF‐16::GFP in LYY15 worms at both 20°C and 25°C. Our results showed that at 20°C, the GFP fluorescence was largely dispersed in the cytosol, whereas at 25°C, the GFP was mainly localized in the nucleus (Figure S13). These results indicate that under the propagation temperature of 25°C, DAF‐16 translocated to the nucleus, thus becoming active in regulating gene expression.

To examine whether DAF‐16 is required for the elevated expression of collagen genes in *npr‐8(ok1439)* worms at 25°C, we performed qRT‐PCR to measure the mRNA levels of three collagen genes (*col‐49*, *col‐77*, and *rol‐1*) in wild‐type, *npr‐8(ok1439)*, and the double mutant *daf‐16(mu86);npr‐8(ok1439)* worms grown at 25°C. Our results showed that the expression of these collagen genes was significantly decreased in the double mutant worms compared to *npr‐8(ok1439)* worms (Figure S14), indicating that *daf‐6* is required for their enhanced expression in *npr‐8(ok1439)* worms. It is worth noting that the expression of these collagen genes in the double mutant worms was higher than that in wild‐type worms (Figure S14), suggesting that additional pathways could be involved in elevating the expression of NPR‐8‐regulated collagen genes in *npr‐8(ok1439)* worms at 25°C.

### 2.6 *npr‐8(ok1439)* animals have a less‐wrinkled cuticle and appear younger than age‐matched wild‐type animals

**FIGURE 4:**
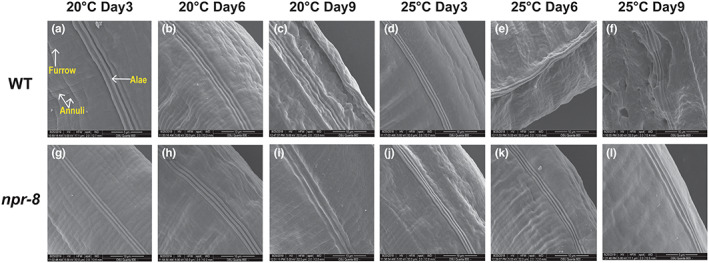
Cuticle of *npr‐8(ok1439)* animals was less wrinkled than that of WT animals at old ages at 20 and 25°C. Adult animals were collected at Days 3, 6, and 9 and imaged using FEI Quanta 600 FEG scanning electron microscope.

During our study, we observed that *npr‐8* mutant animals appeared categorically younger than age‐matched wild‐type animals by exhibiting fewer wrinkles in the cuticle. To investigate this phenomenon in detail, we performed scanning electron microscopy (SEM) to examine the cuticle of wild‐type and *npr‐8(ok1439)* animals at various ages (3‐, 6‐, and 9‐day‐old adults) under the two different propagation temperatures of 20°C and 25°C. Characteristic structural features of the *C. elegans* cuticle include small circumferential furrows separated by broader ridges called annuli and longitudinally oriented ridges called alae (Figure 4a) (Lints & Hall, 2009). Distinct alae are considered an indicator of worm adulthood (Altun & Hall, 2009). Indeed, we observed that 3‐day‐old and 6‐day‐old wild‐type adult animals propagated at 20°C exhibited smooth cuticle with distinct alae structures (Figure 4a,b). At the same temperature, 9‐day‐old wild‐type adults displayed a wrinkled cuticle with distorted alae (Figure 4c), whereas 9‐day‐old *npr‐8(ok1439)* animals maintained distinct alae structures on a smooth surface (Figure 4i). These results demonstrated that there are NPR‐8‐dependent differences in the cuticles of mutant and wild‐type animals. In contrast to what was observed at 20°C, wrinkled cuticles and disintegrated alae were observed among 6‐day‐old wild‐type adults at 25°C (Figure 4e), and by day 9, alae were disrupted (Figure 4f), suggesting that warm temperature speeds up aging. Compared to wild‐type adults, both 6‐day‐old and 9‐day‐old *npr‐8(ok1439)* animals displayed a smooth cuticle with distinct structural morphology at 25°C (Figure 4k,l), indicating younger‐than‐wild‐type physiological age and perhaps better overall health.

## 3 DISCUSSION

As in many other invertebrate and vertebrate animals, temperature has inverse effects on longevity in *C. elegans*. Mechanistically, this inverse relationship is not well understood. In the current study, we have demonstrated that functional loss of NPR‐8 increases *C. elegans* lifespan at 25°C but not at 20°C and 15°C, indicating that NPR‐8 is involved in the longevity response to warm temperature. Further investigation revealed that such regulation is achieved by altering the expression of a subset of collagen genes, and that this regulation is controlled by the NPR‐8‐expressing amphid chemosensory neurons AWB and AWC as well as AFD thermosensory neurons. Evidence in the literature indicates that these neurons may play different roles in NPR‐8‐dependent temperature sensing. On the one hand, AWC neurons are heat sensitive. AWC can sense thermal gradients directly and have been shown to mediate *C. elegans* thermotactic behavior in a way distinct from AFD neurons, a major sensor of temperature and regulator of thermotaxis (Biron et al., 2008). On the other hand, AWB neurons are not heat sensitive but are part of the AFD thermosensory circuit (Mori & Ohshima, 1995). AWB can interact with AFD, AIZ, and RIA neurons in this circuit via chemical synapses. AWB neurons do not directly sense thermal gradients in the physiological range (Mori & Ohshima, 1995), nor can they sense noxious temperatures exceeding 33°C (Liu et al., 2012; Wittenburg & Baumeister, 1999). Together with these findings, our study suggests that NPR‐8 functions redundantly in AWB and AWC neurons to regulate the longevity response to warm temperature, and that AWC neurons likely sense the warm temperature directly while AWB neurons could be part of the AFD thermosensory circuit that can also detect the warm temperature but is independent of AWC‐mediated temperature sensing. Nonetheless, an NPR‐8‐dependent neural‐longevity regulatory mechanism has emerged whereby the nervous system controls the longevity response to warm temperature by regulating collagen expression. However, many details of this regulatory mechanism are lacking. For example, which neurons form a network with AWB, AWC, and AFD neurons to control longevity at warm temperature? What neuroendocrine signaling pathways are used to relay neural signals to non‐neural tissues, since we have previously found that NPR‐8‐regulated collagens are primarily expressed in the cuticle and hypodermal and rectal cells (Sellegounder et al., 2019)? How does collagen expression influence aging? Addressing these questions will provide important mechanistic insights into the temperature‐induced longevity response and define the role of the nervous system in this process.

The inverse relationship between longevity and temperature has long been explained using the rate of living theory, which posits that higher temperatures increase chemical reaction rates, thus speeding up the aging process (Demetrius, 2013). However, recent research has identified specific molecules, cells, and signaling pathways that are involved in the longevity response to temperature, indicating that this response is a regulated process and not simply due to changes in thermodynamics (Keil et al., 2015; Kim et al., 2020). The actual mechanisms behind the inverse relationship seem not quite that clear. On the one hand, our study provides further evidence supporting that the temperature‐induced longevity response is indeed a regulated process. We have identified the neuronal GPCR NPR‐8 and the AWB, AWC, and AFD neurons that function in the *C. elegans* longevity response to warm temperature, indicating that this response is controlled by the nervous system. On the other hand, our RNA‐seq data revealed the enrichment of many genes involved in metabolic/biosynthetic processes in wild‐type animals at 25°C relative to 20°C (Table S2), reflecting increased metabolism at warm temperature. Thus, our data provide a partial molecular basis for the rate of living theory, supporting the idea that there is some scientific truth in this theory. As the rate of living theory and the view of the temperature‐induced longevity response being a regulated process are not mutually exclusive, we propose a hybrid hypothesis in which higher temperatures speed up chemical reactions and the aging process in both thermodynamic and neurally regulated manners in living organisms.

An important finding of our current study is that the expression of NPR‐8‐regulated collagen genes is required for maintaining the lifespan of wild‐type animals at 25°C, and that the expression of these genes contributes to the extended lifespan of *npr‐8(ok1439)* animals at warm temperature (Figure 3). Collagen expression has also been implicated in enhanced resistance to pathogen infection (Sellegounder et al., 2019) and oxidative stress (Ewald et al., 2015). In fact, elevated collagen expression is a common feature of multiple conserved longevity pathways and essentially every longevity intervention (Ewald et al., 2015; Myllyharju & Kivirikko, 2004; Varani et al., 2006). These findings indicate a general role of collagen in aging. Indeed, we observed that *npr‐8* mutant animals with increased collagen expression had a less‐wrinkled cuticle and appeared younger than age‐matched wild‐type animals (Figure 4). Given these younger traits of the mutants, it is paradoxical that *npr‐8(ok1439)* animals had the same lifespan as wild‐type animals at 20°C (Figure 1). We reasoned that in addition to enhanced collagen expression, functional loss of NPR‐8 might cause other unidentified physiological changes in *C. elegans* that negate the positive effect of collagen on aging. However, under stressful conditions, such as pathogen infection (Sellegounder et al., 2019), oxidative stress (Ewald et al., 2015), and warm temperatures (this study), the beneficial effects of elevated collagen gene expression could outweigh other effects of the *npr‐8* mutation, resulting improved survival and longevity. As stress resistance is a critical parameter for assessing healthy lifespan, or healthspan (Bansal et al., 2015), collagen expression likely promotes healthy aging. Therefore, research deciphering how collagen expression promotes longevity may hold the key to the human request for long and healthy life.

## 4 MATERIALS AND METHODS

### 4.1 *Caenorhabditis elegans* strains

*Caenorhabditis elegans* strains were cultured under standard conditions and fed *E. coli* OP50. Wild‐type animals were *C. elegans N2* Bristol. *npr‐8 (ok1439)*, *npr‐8(ok1446)*, *ttx‐1 (p767)* (AFD‐defective mutant), and *col‐179(ok3010)* animals were obtained from the Caenorhabditis Genetics Center (University of Minnesota, Minneapolis, MN) and backcrossed with wild‐type N2 for 3–8 times. JRS30 [*npr‐8(ok1439);col‐179(ok3010)*], LYY11 [*ttx‐1(p767);npr‐8(ok1439)*], LYY12 [*daf‐16(mu86);npr‐8(ok1439)*], LYY13 [*rol‐1p::rol‐1gDNA::gfp*]; LYY14 [*col‐49p::col‐49gDNA::gfp*], LYY15 [*npr‐8(ok1439);zls356*], JRS17 [*npr‐8p(2 kb)::npr‐8 gDNA::gfp*] (native NPR‐8 rescue strain), JRS18 [*str‐1p(1.3 kb)::npr‐8 gDNA::gfp*] (NPR‐8 rescue in AWB neurons), JRS19 [*str‐2p(2 kb)::npr‐8 gDNA::gfp*] (NPR‐8 rescue in AWC neurons), JRS20 [*trx‐1p(1.1 kb)::npr‐8 gDNA::gfp*] (NPR‐8 rescue in ASJ neurons), and transcriptional reporter strain *npr‐8p::gfp* were generated using standard genetic techniques (Sellegounder et al., 2019).

### 4.2 Lifespan assay

Wild‐type and mutant worms were maintained at 20°C and fed *E. coli* OP50 on NGM medium. For temperature‐specific assays at 15 and 25°C, worm strains were maintained for at least two generations at the respective temperature before being used for egg synchronization. For egg synchronization, 3 × 20 gravid adult worms per strain were used. The time window of egg synchronization was set for 1 h, during which all animals were maintained at the respective assay temperature (15°C, 20°C, or 25°C). *E. coli OP50* lawn was prepared by spreading a 50‐μL drop of an overnight culture of bacteria on the NGM medium (3.5‐cm petri plates). Plates were incubated at 37°C for 16 hrs and then cooled down at room temperature for at least 1 h before seeding with synchronized worms. 3 × 20 synchronized worms for each group were transferred onto the live *E. coli* OP50 plates. Worms were counted and transferred to fresh, live *E. coli* OP50 culture daily until death. All assays were performed in triplicates for each strain.

### 4.3 Heat stress assay

Wild‐type and *npr‐8 (ok1439)* gravid adult animals were bleached to isolate eggs. Synchronized L1 worms were plated on *E. coli* OP50 and maintained at 20°C until they reached adulthood. One‐day‐old adult worms were then transferred to 35°C on NGM medium and scored for survival every 2 h until all worms were dead.

### 4.4 RNA isolation

To gain molecular insights into the NPR‐8‐dependent longevity response to temperature, we employed RNA‐seq to compare gene expression in young and old wild‐type and *npr‐8(ok1439)* animals propagated at different temperatures. To this end, we collected five replicates of eight groups of RNA samples (young (1‐day‐old) and 9‐day‐old wild‐type and *npr‐8(ok1439)* adults propagated at 20 and 25°C) (Table 1). The temperature‐specific worm cultivation and synchronization steps were performed as described in the lifespan assay section. A portion of synchronized adult animals were collected at the age of 1 day old, and the remaining animals were grown till Day 9 before collection. These animals were washed every day using M9 buffer, and the adult animals were filtered using cell strainer (40‐μm nylon filter, Falcon) and transferred to *E. coli* OP50‐seeded NGM plates. Worms were washed and collected with M9 buffer then snap‐frozen in the TRIzol reagent (Thermo Fisher Scientific) and stored at −80°C until RNA isolation. RNA was extracted using the QIAzol lysis reagent (Qiagen) and purified with the RNeasy Plus Universal Kit (Qiagen).

### 4.5 RNA sequencing

Total RNA samples were obtained as described above and submitted to the WSU Genomics Core for RNA‐seq analysis. RNA‐seq and related bioinformatic analyses were performed following our published protocols with modifications (Sellegounder et al., 2019). Specifically, after alignment of FASTQ files to the *C. elegans* reference genome (ce10, UCSC) using HISAT2 (Kim et al., 2015), gene expression quantification and differential expression were analyzed using Cuffquant and Cuffdiff, respectively (Trapnell et al., 2012). Principal component analysis (PCA) was done following our previously described protocol (Wang et al., 2022). Volcano plot analysis was done following the protocol of EnhancedVolcano (Blighe et al., 2022). To identify the most highly and significantly changed genes, *p‐*value cut‐off and log2FoldChange cut‐off were arbitrarily set at 10e‐100 and 5, respectively. The raw sequence data (FASTQ files) were deposited in the National Center for Biotechnology Information (NCBI) Sequence Read Archive (SRA) database through the Gene Expression Omnibus (GEO). The processed gene quantification files and differential expression files were deposited in the GEO. All of these data can be accessed through the GEO with the accession number GSE186202.

### 4.6 Fluorescence imaging

Fluorescence microscopy imaging of *C. elegans* was performed as previously described (Sellegounder et al., 2019). Briefly, animals were anesthetized with 30 mM sodium azide, placed on a freshly prepared 2% agarose pad, and covered with a 1 mm glass coverslip. Images were taken using a Zeiss Axio Imager M2 fluorescence stereomicroscope with a 40× objective lens and a 1.6× Tube lens.

### 4.7 DAF‐16 nuclear localization assay

To analyze the subcellular localization of DAF‐16 in *npr‐8(ok1439)* worms, *npr‐8(ok1439)* worms were crossed with the DAF‐16::GFP reporter strain TJ356 (genotype: *zls356* [*daf‐16p::daf‐16a/b::GFP + rol‐6(su1006)*], (Henderson & Johnson, 2001)) to create strain LYY15 [*npr‐8(ok1439);zls356*] that expresses DAF‐16::GFP in a *npr‐8* mutant background. LYY15 worms were maintained at 20°C. To image DAF‐16::GFP at both 20 and 25°C, LYY15 worms were propagated under these two temperatures for at least two generations, synchronized using the bleaching method, and grown to the L4 stage. L4 worms were anesthetized with 30 mM sodium azide, transferred onto a freshly prepared 2% agarose pad, and then covered with a 1‐mm glass coverslip. Images were taken using a Zeiss Axio Imager M2 fluorescence stereomicroscope with a 10× objective lens.

### 4.8 Plasmid construction and overexpression strain generation

Transgenic animals were generated following our published protocol (Sellegounder et al., 2019). Briefly, to generate *rol‐1* overexpression strain, the *rol‐1* promotor including 5′UTR (2030 bp) and its coding region including introns and 3′UTR (3776 bp) were PCR amplified from the genomic DNA of 1‐day‐old wild‐type *C. elegans* adults and cloned into the plasmid *pPD95.77* between *Pst I* and *Xma I* sites to generate *pPSN01*. A mixture of *pPSN01* (5 ng/μL), co‐injection marker *pCFJ104* (*myo‐3::mCherry*) (10 ng/μL), carrier DNA *pNU1254* (*HygR*) (15 ng/μL), and salmon testes DNA (STD, filler DNA) (70 ng/μL) were injected into wild‐type animals to create the *rol‐1* overexpression strain LYY13.

To generate *col‐49* overexpression strain, the *col‐49* promotor including 5′UTR (1353 bp) and its coding region including introns and 3′UTR (1238 bp) were PCR amplified from the genomic DNA of 1‐day‐old wild‐type *C. elegans* adults and cloned into *pPD95.77* between *Sph I* and *Xma I* sites to generate the plasmid *pPSN03*. A mixture of *pPSN03* (5 ng/μL), co‐injection marker *pCFJ104* (*myo‐3::mCherry*) (10 ng/μL), carrier DNA *pNU1254* (*HygR*) (15 ng/μL), and STD (70 ng/μL) were injected into wild‐type animals to create the *col‐49* overexpression strain LYY14.

### 4.9 Scanning electron microscopy (SEM) analysis

To determine age‐ and temperature‐dependent structural changes in the cuticle, we performed SEM to examine wild‐type and *npr‐8 (ok1439)* animals at different ages (3‐, 6‐, and 9‐day‐old adults) propagated at 20°C or 25°C. Temperature‐specific worm maintenance and synchronization were the same as described in the lifespan assay. Sample preparation and image acquisition were performed as we previously described (Sellegounder et al., 2019).

### 4.10 RNA interference

The collagen genes *col‐77*, *col‐49*, *col‐139*, and *rol‐1* were individually silenced using RNAi in wild‐type and *npr‐8 (ok1439)* animals at 25°C. The temperature‐specific worm cultivation and synchronization steps were performed as described in the above lifespan assay section. The RNAi protocol was performed as we previously described (Sellegounder et al., 2019). The impact of collagen gene silencing on *C. elegans* lifespan was assessed using lifespan assays.

### 4.11 Statistical analysis

For *C. elegans* lifespan assays, animal survival was plotted as a non‐linear regression curve using the PRISM computer program (version 9; GraphPad Software, Inc.). Lifespan curves were considered different than the appropriate controls when *p*‐values were <0.05. Prism uses the product limit or Kaplan–Meier method to calculate survival fractions and the logrank test (equivalent to the Mantel‐Heanszel test) to compare survival curves. All of the experiments were repeated at least three times, unless otherwise indicated.

The median lifespan and the percentage increase in median lifespan relative to controls for all lifespan assays performed in this study were calculated using the PRISM computer program. The median lifespan was calculated based on the time of death for 50% of the total worm population (Machin et al., 2006). The percentage increase in median lifespan was calculated by dividing the median lifespan of a particular strain by that of its control strain and converting to a percentage. The results were summarized in Table S1.

## AUTHOR CONTRIBUTIONS

Sankara Naynar Palani, Durai Sellegounder, Phillip Wibisono, and Yiyong Liu designed and performed experiments and analyzed data. Sankara Naynar Palani and Yiyong Liu wrote the paper.

## FUNDING INFORMATION

This work was supported by the Department of Translational Medicine and Physiology, Elson S. Floyd College of Medicine, WSU‐Spokane (Startup to Y.L.). The funder had no role in study design, data collection and interpretation, or the decision to submit the work for publication.

## CONFLICT OF INTEREST STATEMENT

The authors declare that they have no competing interests.

## Supporting information


Fig. S1

Table S1

Table S2

Table S3

Table S4

Table S5

Table S6

Table S7

Table S8

Table S9

Table S10

Table S11

Table S12

Table S13

Table S14

Table S15

Table S16

Table S17

Table S18

Table S19

Table S20

## Data Availability

The RNA sequencing data have been deposited in NCBI's Sequence Read Archive (SRA) database through the Gene Expression Omnibus (GEO). Processed gene quantification files and differential expression files have been deposited in GEO. All of these data can be accessed through the GEO with the accession number GSE186202. All data needed to evaluate the conclusions in the paper are present in the paper and/or the Supporting Information. Additional data are available from the authors upon request. The *C. elegans* strains and plasmids constructed by us and the primer sequences used for their construction are available upon request.
