# Supplementary material for: The longevity response to warm temperature is neurally controlled via the regulation of collagen genes
Source: Aging Cell. 2023 Mar 9;22(5):e13815. doi: 10.1111/acel.13815 (PMC10186602; doi:10.1111/acel.13815)
Supplement: Supplementary file 1 — Fig. S1 [file ACEL-22-e13815-s014.pdf]

Supplementary Figures

Fig. S1

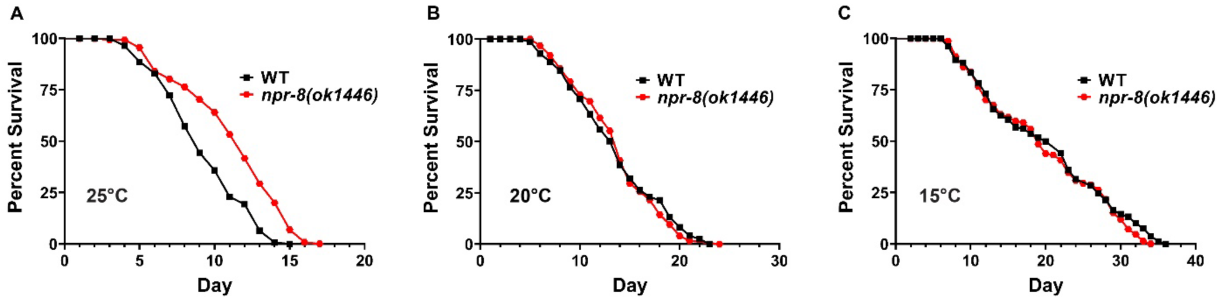

Fig. S1. *npr-8(ok1446)* animals live longer than WT animals at 25°C but not at 20°C or 15°C. WT and *npr-8(ok1446)* animals were grown on *E. coli* strain OP50 at 25°C **(A)**, 20°C **(B)**, or 15°C **(C)**, and scored for survival over time. The graphs are the combined results of three independent experiments. Each experiment included $n = 60$ adult animals per strain. p-values represent the significance level of the mutants relative to the WT, $p < 0.0001$ in (A), $p = 0.8114$ in (B), and $p = 0.2202$ in (C).

Fig. S2

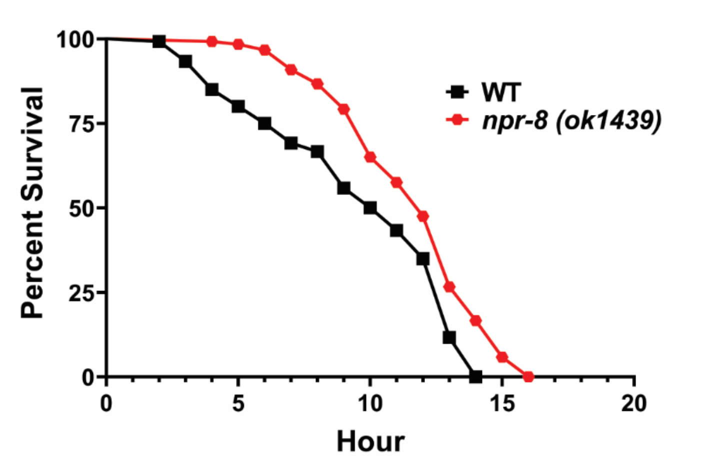

Fig. S2. ***npr-8(ok1439)* animals were more resistant to heat stress at 35°C than wild-type animals.** One-day-old adult wild-type and *npr-8(ok1439)* animals were transferred from 20°C to 35°C and scored for survival over time. The graphs are the combined results of two independent experiments. Each experiment included n = 60 adult animals per strain. p-value < 0.0001

Fig. S3

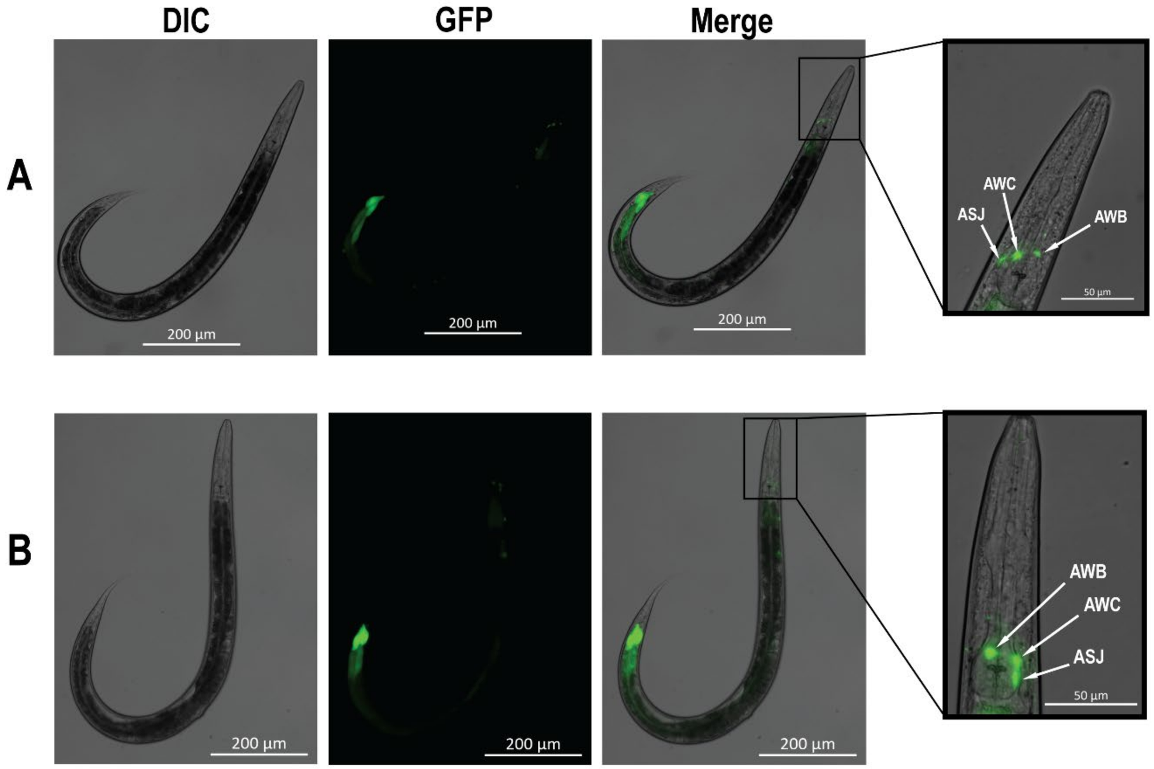

Fig. S3. NPR-8 is expressed in the amphid sensory neurons AWB, ASJ, and AWC, as well as possibly in the lower gut region under both the propagation temperatures of 20ºC and 25ºC. Young-adult *npr-8p::gfp* animals were grown at 20ºC (A) or 25ºC (B), anesthetized with 30 mM sodium azide, placed on a freshly prepared 2% agarose pad, and covered with a 1 mm glass coverslip. Images were taken using a Zeiss Axio Imager M2 fluorescence stereomicroscope with a 40x objective lens and a 1.6x Tube lens. The fluorescence in the lower gut region could be background fluorescence conferred by the backbone of the plasmid *pPD95.77* but could also result from GFP expression driven by the *npr-8* promoter.

Fig. S4

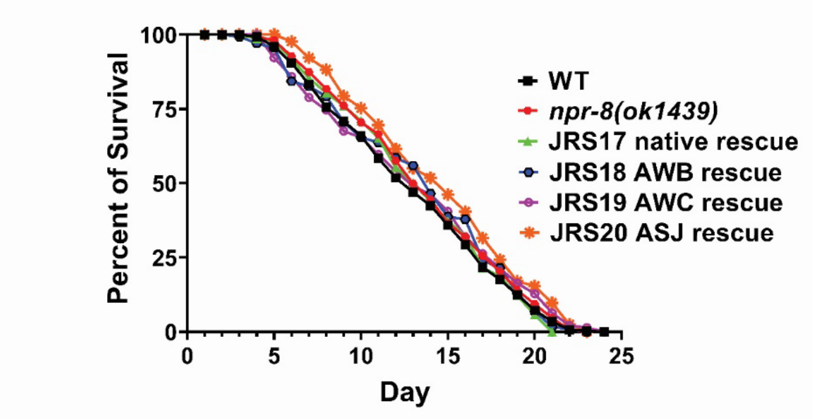

Fig. S4. NPR-8 rescue animals exhibited lifespan similar to that of wild-type animals at 20°C. WT, *npr-8 (ok1439)*, and rescue animals were grown at 20°C and scored for survival over time. JRS17, *npr-8* expression restored in *npr-8(ok1439)* under its native promoter; JRS18, *npr-8* expression rescued in AWB neurons; JRS19, *npr-8* expression rescued in AWC neurons; and JRS20, *npr-8* expression rescued in ASJ neurons. The graphs are the combined results of three independent experiments. Each experiment included $n = 60$ adult animals per strain. *p*-values are relative to WT: *npr-8 (ok1439)*, $p = 0.3424$; JRS17, $p = 0.9600$; JRS18, $p = 0.6348$; JRS19, $p = 0.9119$; JRS20, $p = 0.0066$.

Fig. S5

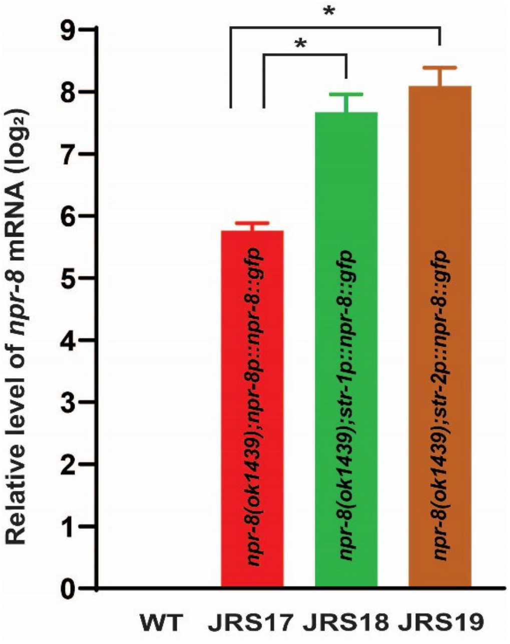

Fig. S5. The levels of *npr-8* expression were higher in JRS18 and JRS19 worms than that in JRS17 worms. qRT-PCR was performed to measure *npr-8* mRNA levels in WT, JRS17 [*npr-8(ok1439);npr-8p::npr-8::gfp*], JRS18 [*npr-8(ok1439);str-1p::npr-8::gfp*], and JRS19 [*npr-8(ok1439);str-2p::npr-8::gfp*] worms propagated at 25°C. In all assays, pan-actin was used as an internal control, and *npr-8* expression was normalized against WT. Bars represent mean ± SEM, and values are the average of three independent experiments. An asterisk (*) denotes a significant difference ($p < 0.0001$) in expression between JRS17 and JRS18 or between JRS17 and JRS19.

Fig. S6

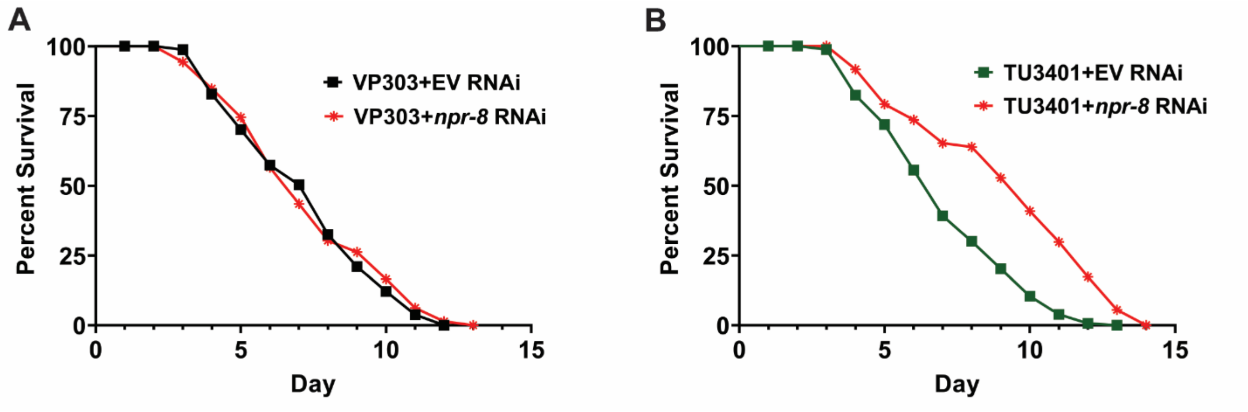

Fig. S6. Inactivation of *npr-8* in neurons, but not in the intestine, extends worm lifespan at 25°C. VP303 animals (capable of intestine-specific RNAi) **(A)** and TU3401 animals (capable of neuron-specific RNAi) **(B)** were grown on dsRNA for empty vector (EV) or for *npr-8* at 25°C and scored for survival over time. The graphs are the combined results of three independent experiments. Each experiment included n = 60 adult animals per strain. p-values are $p = 0.5544$ in (A) and $p < 0.0001$ in (B).

Fig. S7

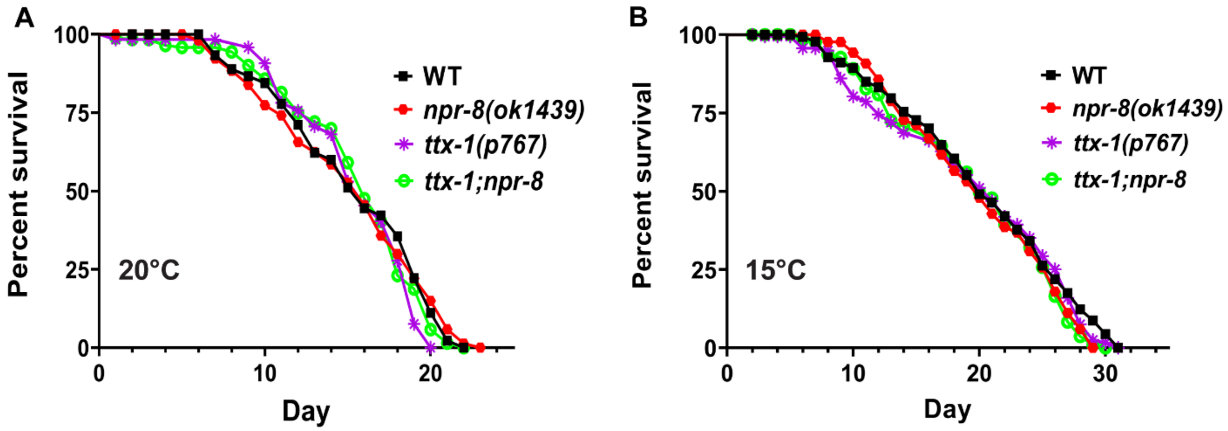

Fig. S7. Functional loss of NPR-8 and/or AFD neurons had no effects on longevity under the propagation temperature 20°C or 15°C. WT, *npr-8(ok1439)*, *ttx-1(p767)*, *ttx-1;npr-8(lyy11)* animals were grown at 20°C **(A)** or 15°C **(B)** and scored for survival over time. The graphs are the combined results of three independent experiments. Each experiment included $n = 60$ adult animals per strain. In (A), p-values are relative to WT: *npr-8(ok1439)*, $p = 0.2186$; *ttx-1(p767),* $p = 0.3196$; *ttx-1;npr-8,* $p = 0.6794$. In (B), p-values are relative to WT: *npr-8(ok1439)*, $p = 0.1633$; *ttx-1(p767),* $p = 0.4349$; *ttx-1;npr-8,* $p = 0.1313$.

Fig. S8

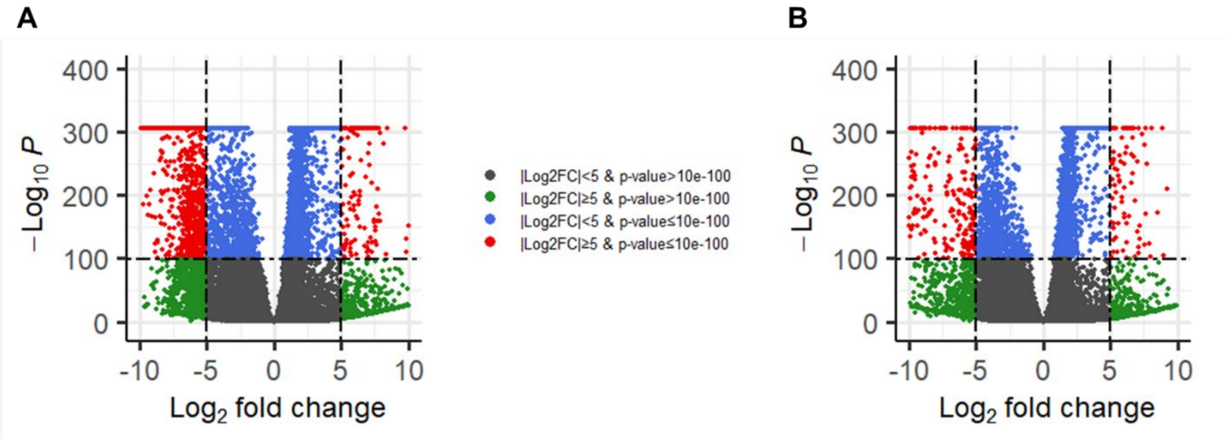

Fig. S8. The most highly and significantly changed genes impacted by age or temperature were determined by volcano plots. The volcano plots were generated following the protocol of EnhancedVolcano (Blighe et al. 2022 https://github.com/kevinblighe/EnhancedVolcano). **(A)** was made from the dataset for assessing the age effects (9-day-old vs. 1-day-old wild-type adults grown at 20°C), and **(B)** was made from the dataset for assessing the temperature effects (1-day-old wild-type adults grown at 20°C vs. 25°C). *p*-value cut-off and log2FoldChange cut-off were arbitrarily set at 10e-100 and 5, respectively. The most highly and significantly changed genes were listed in Table S12.

Fig. S9

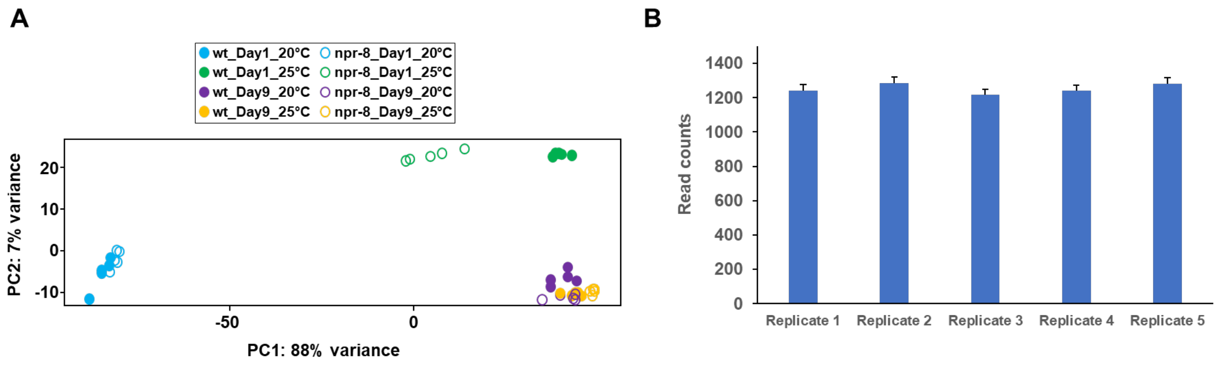

Fig. S9. Principal component analysis (PCA) of RNA-seq data. (A) PCA of RNA-seq data from 40 samples (five replicates of eight groups of samples listed in Table 1) was performed using log2(cpm+1) values. **(B)** The mean read counts of all genes detected in each of the five replicates in group 1 were plotted. The values were compared between replicates using the two-sample *t*-test, and no significant difference was found between any two replicates. Error bars represent the standard error of the mean (SEM).

Fig. S10

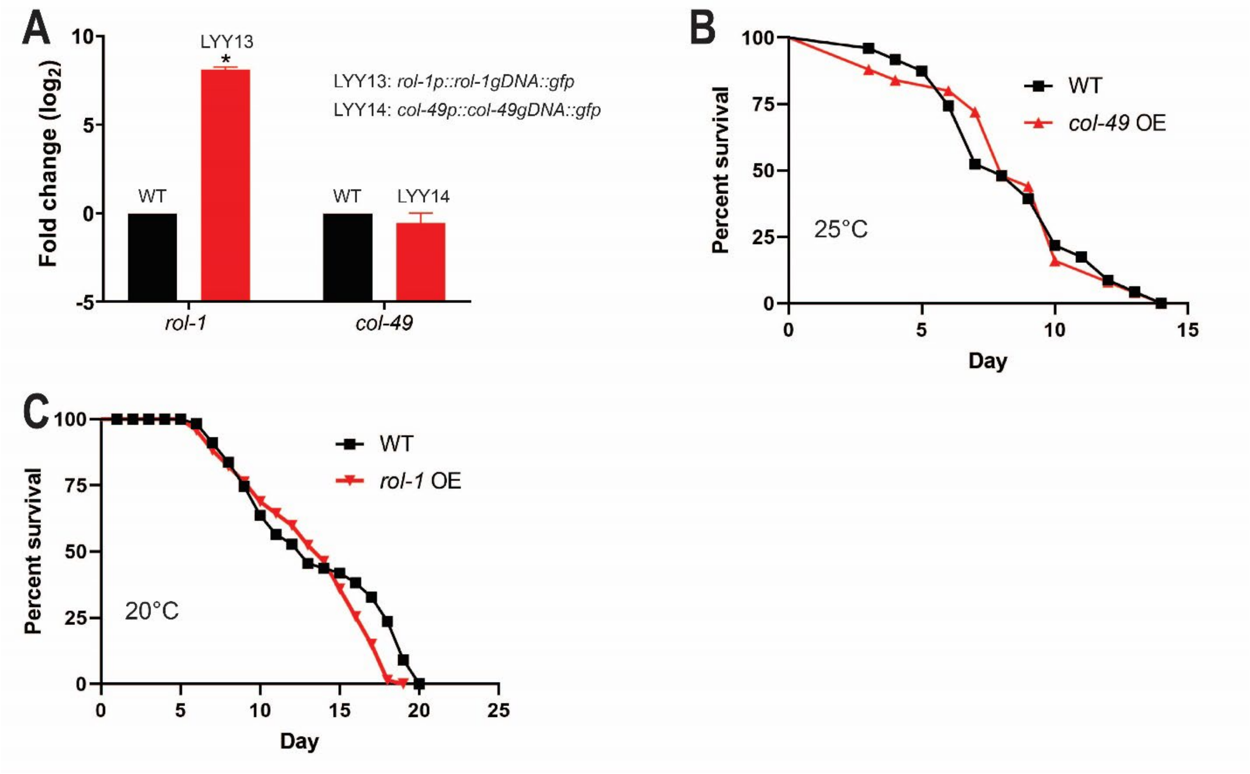

Fig. S10. The roles of NPR-8-regulated collagen genes in longevity were assessed using collagen gene overexpression and lifespan assays. (A) qRT-PCR analysis was performed to measure *rol-1* expression in WT worms and the transgenic LYY13 (*rol-1p::rol-1gDNA::gfp*) worms and to measure *col-49* expression in WT worms and the transgenic LYY14 (*col-49p::col-49gDNA::gfp*) worms. The graphs are combined results of three independent experiments. Bars represent mean ± SEM. An asterisk (*) denotes a significant difference ($p < 0.0001$) in expression between WT and LYY13 worms. **(B)** WT and *col-49* overexpressing strain LYY14 were grown at 25°C and scored for survival over time. The graphs are the combined results of three independent experiments. Each experiment included $n = 60$ adult animals per strain. The p-value between the two strains is 0.845. **(C)** WT and *rol-1* overexpressing strain LYY13 were grown at 20°C and scored for survival over time. The graphs are the combined results of three independent experiments. Each experiment included $n = 60$ adult animals per strain. The p-value between the two strains is 0.4107.

Fig. S11

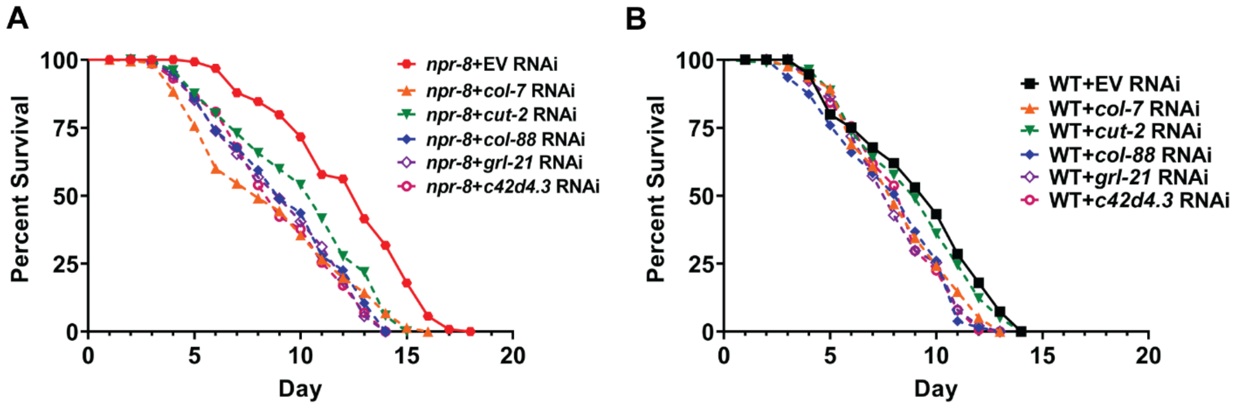

Fig. S11. Genes upregulated in *npr-8(ok1439)* animals at 25°C but not at 20°C in Day 1 contributed to the mutants' extended lifespan at 25°C. *npr-8(ok1439)* animals **(A)** and WT animals **(B)** were grown on dsRNA for empty vector (EV) or for *col-7*, *cut-2*, *col-88*, *grl-21*, *or C42D4.3* at 25°C and scored for survival over time. The graphs are the combined results of three independent experiments. Each experiment included $n = 60$ adult animals per strain. *p*-values are relative to *npr-8*+EV in (A): *npr-8+col-7*, p <0.0001; *npr-8+cut2*, p <0.0001; *npr-8+col-88*, p < 0.0001; *npr-8+grl-21*, p <0.0001; and *npr-8+C42D4.3*, p < 0.0001. *p*-values are relative to WT+EV in (B): WT+*col-7, p* = 0.0004; WT+*cut 2, p* = 0.3035; WT+*col-88, p* < 0.0001; WT+ *grl-21, p* < 0.0001; WT+*C42D4.3, p*<0.0001.

Fig. S12

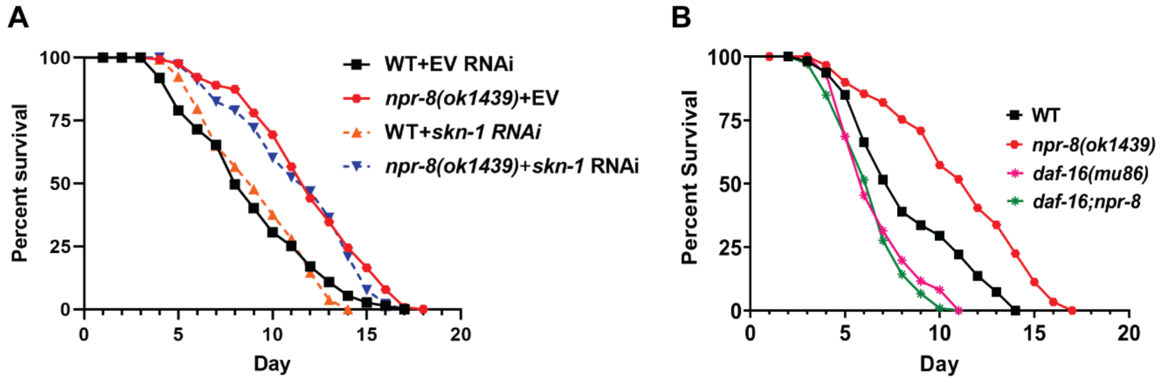

Fig. S12. SKN-1 is not involved in the NPR-8-dependent longevity response to warm temperature, while DAF-16 is. (A) *npr-8(ok1439)* animals and WT animals were grown on dsRNA for empty vector (EV) or for *skn-1* at 25°C and scored for survival over time. The graphs are the combined results of three independent experiments. Each experiment included $n = 60$ adult animals per strain. The p-value between *npr-8(ok1439)*+EV and *npr-8(ok1439)+skn-1* is 0.1312, and the p-value between WT+EV and WT+*skn-1* is 0.8456. **(B)** WT, *npr-8(ok1439), daf-16* (*mu86*), and *daf-16;npr-8* animals were grown at 25°C and scored for survival over time. The graphs are the combined results of two independent experiments. Each experiment included $n =$ 60 adult animals per strain. The p-values relative to *npr-8(ok1439)* are: *daf-16 (mu86)*, $p <$ 0.0001 and *daf-16;npr-8*, $p < 0.0001$. The p-values relative to WT are: *daf-16 (mu86)*, $p <$ 0.0001 and *daf-16;npr-8*, $p < 0.0001$.

Fig. S13

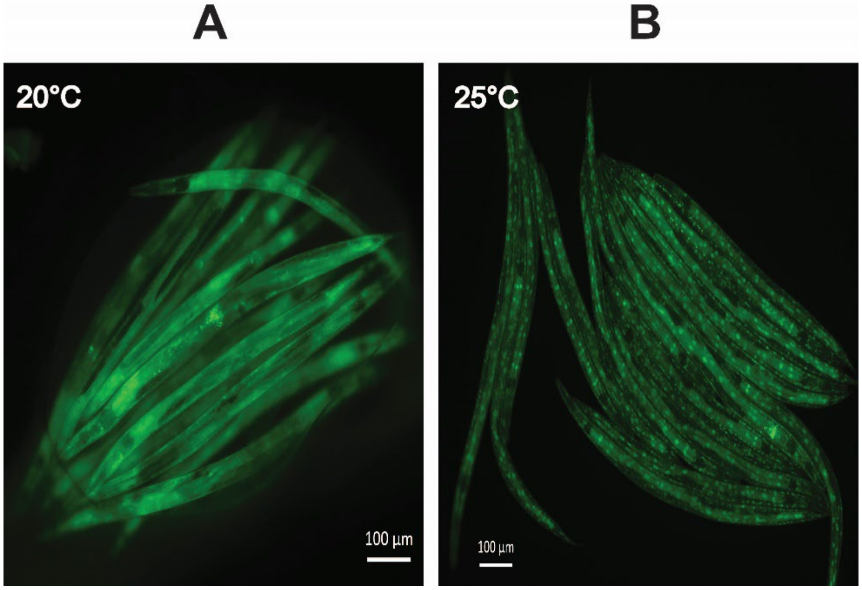

Fig. S13. DAF-16 translocated to the nucleus under the propagation temperature of 25ºC. LYY15 [*npr-8(ok1439);zls356*] worms that express DAF-16::GFP in a *npr-8* mutant background were propagated at 20ºC **(A)** or 25ºC **(B)** for at least two generations, synchronized using the bleaching method, and grown to the L4 stage. L4 worms were anesthetized with 30mM sodium azide, placed on a 2% agarose pad and covered with a 1-mm glass coverslip, and imaged using a Zeiss Axio Imager M2 fluorescence stereomicroscope with a 10X objective lens.

Fig. S14

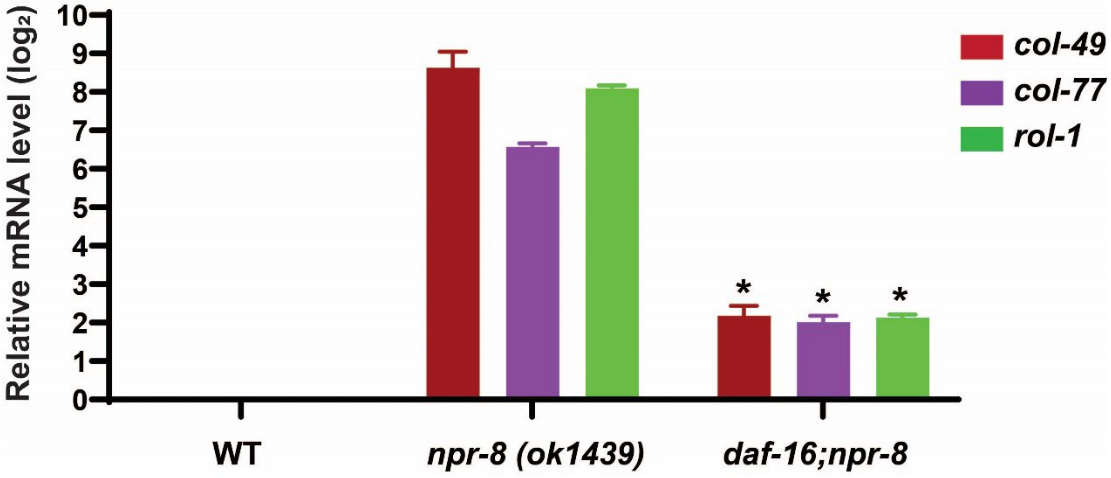

Fig. S14. DAF-16 is required for the enhanced expression of collagen genes in *npr-8(ok1439)* worms at 25ºC. qRT-PCR analysis were performed to measure the expression of collagen genes (*col-49*, *col-77*, and *rol-1*) in WT, *npr-8(ok1439)*, and *daf-16(mu86);npr-8(ok1439)* worms propagated at 25ºC. In all assays, pan-actin was used as an internal control, and collagen gene expression was normalized against WT. Bars represent mean ± SEM, and values are the average of three independent experiments. Asterisks (*) denote significant difference ($p <$ 0.0001) in expression between *npr-8(ok1439)* and *daf-16(mu86);npr-8(ok1439)* worms.
