# Supplementary material for: The longevity response to warm temperature is neurally controlled via the regulation of collagen genes
Source: Aging Cell. 2023 Mar 9;22(5):e13815. doi: 10.1111/acel.13815 (PMC10186602; doi:10.1111/acel.13815)
Supplement: Supplementary file 2 — Table S1 [file ACEL-22-e13815-s006.docx]

**Table S1. Median lifespan and percentage increase in median lifespan of all lifespan assays performed in this study**

| Figure | Strain | Assay Temperature | Median Lifespan  (days) | % Increase in Median Lifespan |
| --- | --- | --- | --- | --- |
| Fig.1A | WT | 25°C | 10 | control |
|  | *npr-8 (ok1439)* |  | 13 | 30.0% |
| Fig.1B | WT | 20°C | 14 | control |
|  | *npr-8 (ok1439)* |  | 16 | 14.3% |
| Fig.1C | WT | 15°C | 25 | control |
|  | *npr-8 (ok1439)* |  | 23 | -8.0% |
| Fig.1D | WT | 25°C | 9 | control |
|  | *npr-8 (ok1439)* |  | 11 | 22.3% |
|  | JRS17 |  | 10 | 11.2% |
| Fig.2A | WT | 25°C | 9 | control |
|  | *npr-8 (ok1439)* |  | 11 | 22.2% |
|  | JRS18 |  | 6 | -33.4% |
|  | JRS19 |  | 8 | -11.1% |
|  | JRS20 |  | 11 | 22.3% |
| Fig.2B | WT | 25°C | 7 | control |
|  | *npr-8 (ok1439)* |  | 9 | 28.5% |
|  | *ttx-1 (p767)* |  | 5 | -28.6% |
|  | *ttx-1;npr-8* |  | 7 | 0.0% |
| Fig.3A | *npr-8 (ok1439)* + EV RNAi | 25°C | 10 | control |
|  | *npr-8 (ok1439)* + *col-49* RNAi |  | 7 | -30.0% |
|  | *npr-8 (ok1439)* + *col-77* RNAi |  | 6 | -40.0% |
|  | *npr-8 (ok1439)* + *col-139* RNAi |  | 9 | -10.0% |
|  | *npr-8 (ok1439)* + *rol-1* RNAi |  | 9 | -10.0% |
| Fig.3B | WT + EV RNAi | 25°C | 8 | control |
|  | WT + *col-49* RNAi |  | 7 | -12.5% |
|  | WT + *col-77* RNAi |  | 8 | 0.0% |
|  | WT + *col-139* RNAi |  | 9 | 12.5% |
|  | WT + *rol-1* RNAi |  | 8 | 0.0% |
| Fig.3C | WT | 25°C | 9 | control |
|  | *col-179 (ok3010)* |  | 7 | -22.2% |
|  | *npr-8 (ok1439)* |  | 11 | 22.2% |
|  | *col-179;npr-8* |  | 7 | -22.2% |
| Fig.3D | WT | 25°C | 6 | control |
|  | *rol-1* OE |  | 7 | 16.7% |

| Fig.S1A | WT | 25°C | 9 | control |
| --- | --- | --- | --- | --- |
|  | *npr-8 (ok1446)* |  | 12 | 33.3% |
| Fig.S1B | WT | 20°C | 11 | control |
|  | *npr-8 (ok1446)* |  | 12 | 9.9% |
| Fig.S1C | WT | 15°C | 20 | control |
|  | *npr-8 (ok1446)* |  | 19 | -5.0% |
| Fig.S3 | WT | 20°C | 13 | control |
|  | *npr-8 (ok1439)* |  | 13 | 0.0% |
|  | JRS17 |  | 13 | 0.0% |
|  | JRS18 |  | 14 | 7.7% |
|  | JRS19 |  | 12 | -7.7% |
|  | JRS20 |  | 15 | 15.4% |
| Fig.S4A | VP303 + EV RNAi | 25°C | 8 | control |
|  | VP303 + *npr-8* RNAi |  | 7 | -12.5% |
| Fig.S4B | TU3401 + EV RNAi | 25°C | 7 | control |
|  | TU3401 + *npr-8* RNAi |  | 10 | 42.9% |
| Fig.S5A | WT | 20°C | 16 | control |
|  | *npr-8 (ok1446)* |  | 16 | 0.0% |
|  | *ttx-1 (p767)* |  | 16 | 0.0% |
|  | *ttx-1;npr-8* |  | 16 | 0.0% |
| Fig.S5B | WT | 15°C | 20 | control |
|  | *npr-8 (ok1446)* |  | 20 | 0.0% |
|  | *ttx-1 (p767)* |  | 21 | 5.0% |
|  | *ttx-1;npr-8* |  | 20 | 0.0% |
| Fig.S8B | WT | 25°C | 8 | control |
|  | *col-49* OE |  | 8 | 0.0% |
| Fig.S8C | WT | 20°C | 13 | control |
|  | *rol-1* OE |  | 14 | 7.7% |
| Fig.S9A | *npr-8 (ok1439)* + EV RNAi | 25°C | 13 | control |
|  | *npr-8 (ok1439)* + *col-7* RNAi |  | 8 | -38.5% |
|  | *npr-8 (ok1439)* + *cut-2* RNAi |  | 11 | -15.4% |
|  | *npr-8 (ok1439)* + *col-88* RNAi |  | 9 | -30.8% |
|  | *npr-8 (ok1439)* + *grl-21* RNAi |  | 9 | -30.8% |
|  | *npr-8 (ok1439)* + *c42d4.3* RNAi |  | 9 | -30.8% |

| Fig.S9B | WT + EV RNAi | 25°C | 10 | control |
| --- | --- | --- | --- | --- |
|  | WT + *col-7* RNAi |  | 8 | -20.0% |
|  | WT + *cut-2* RNAi |  | 9 | -10.0% |
|  | WT + *col-88* RNAi |  | 9 | -10.0% |
|  | WT + *grl-21* RNAi |  | 8 | -20.0% |
|  | WT + *c42d4.3* RNAi |  | 9 | -10.0% |
| Fig.S10A | *WT* + EV RNAi | 25°C | 8 | control |
|  | *WT* + *skn-1* RNAi |  | 9 | 12.5% |
|  | *npr-8 (ok1439)* + EV RNAi |  | 12 | control |
|  | *npr-8 (ok1439)* + *skn-1* RNAi |  | 12 | 0.0% |
| Fig.S10B | *WT* | 25°C | 8 | control |
|  | *daf-16 (mu86)* |  | 6 | -25.0% |
|  | *npr-8 (ok1439)* |  | 12 | 50.0% |
|  | *daf-16;npr-8* |  | 7 | -12.5% |
| Fig.S11 | WT | 35°C | 10.5 (hr) | control |
|  | *npr-8 (ok1439)* |  | 12 (hr) | 14.3% |
