# Supplementary material for: The longevity response to warm temperature is neurally controlled via the regulation of collagen genes
Source: Aging Cell. 2023 Mar 9;22(5):e13815. doi: 10.1111/acel.13815 (PMC10186602; doi:10.1111/acel.13815)
Supplement: Supplementary file 8 — Table S7 [file ACEL-22-e13815-s007.docx]

**Table S7. Attenuated molecular functions in 9-day-old wild-type animals grown at 20°C relative to 1-day-old adult animals (RNA-seq analysis with 5% FDR)**

**(A) Attenuated molecular functions**

| GO term | Description | P-value^#^ | FDR q-value* | Enrichment (N, B, n, b)^§^ |
| --- | --- | --- | --- | --- |
| GO:0042302 | structural constituent of cuticle | 1.85E-29 | 4.79E-26 | 2.56 (11216,145,3227,107) |
| GO:0022857 | transmembrane transporter activity | 1.53E-18 | 1.97E-15 | 1.50 (11216,769,3227,331) |
| GO:0005215 | transporter activity | 3.53E-18 | 3.03E-15 | 1.48 (11216,804,3227,342) |
| GO:0022803 | passive transmembrane transporter activity | 1.20E-16 | 7.71E-14 | 1.77 (11216,309,3227,157) |
| GO:0015267 | channel activity | 1.20E-16 | 6.17E-14 | 1.77 (11216,309,3227,157) |
| GO:0005216 | ion channel activity | 5.30E-16 | 2.28E-13 | 1.81 (11216,267,3227,139) |
| GO:0022838 | substrate-specific channel activity | 7.73E-16 | 2.85E-13 | 1.80 (11216,273,3227,141) |
| GO:0004721 | phosphoprotein phosphatase activity | 1.01E-14 | 3.27E-12 | 1.99 (11216,168,3227,96) |
| GO:0004725 | protein tyrosine phosphatase activity | 3.94E-14 | 1.13E-11 | 2.34 (11216,89,3227,60) |
| GO:0022836 | gated channel activity | 4.14E-14 | 1.07E-11 | 1.97 (11216,166,3227,94) |
| GO:0015318 | inorganic molecular entity transmembrane transporter activity | 4.51E-14 | 1.06E-11 | 1.56 (11216,464,3227,208) |
| GO:0022839 | ion gated channel activity | 1.01E-13 | 2.17E-11 | 1.96 (11216,163,3227,92) |
| GO:0015075 | ion transmembrane transporter activity | 4.39E-13 | 8.72E-11 | 1.51 (11216,498,3227,217) |
| GO:0016791 | phosphatase activity | 1.17E-12 | 2.16E-10 | 1.75 (11216,241,3227,121) |
| GO:0005261 | cation channel activity | 1.21E-11 | 2.08E-09 | 1.88 (11216,161,3227,87) |
| GO:0022834 | ligand-gated channel activity | 1.91E-11 | 3.08E-09 | 2.01 (11216,121,3227,70) |
| GO:0015276 | ligand-gated ion channel activity | 1.91E-11 | 2.90E-09 | 2.01 (11216,121,3227,70) |
| GO:0042578 | phosphoric ester hydrolase activity | 2.45E-10 | 3.51E-08 | 1.62 (11216,273,3227,127) |
| GO:0008324 | cation transmembrane transporter activity | 8.41E-10 | 1.14E-07 | 1.54 (11216,324,3227,144) |
| GO:0046873 | metal ion transmembrane transporter activity | 8.42E-10 | 1.09E-07 | 1.70 (11216,205,3227,100) |
| GO:0140096 | catalytic activity, acting on a protein | 1.59E-09 | 1.96E-07 | 1.26 (11216,1215,3227,440) |
| GO:0004715 | non-membrane spanning protein tyrosine kinase activity | 1.89E-09 | 2.21E-07 | 2.53 (11216,44,3227,32) |
| GO:0004672 | protein kinase activity | 2.88E-09 | 3.23E-07 | 1.48 (11216,391,3227,166) |
| GO:0030594 | neurotransmitter receptor activity | 7.28E-09 | 7.83E-07 | 1.91 (11216,109,3227,60) |
| GO:0022890 | inorganic cation transmembrane transporter activity | 8.25E-09 | 8.51E-07 | 1.54 (11216,294,3227,130) |
| GO:0015077 | monovalent inorganic cation transmembrane transporter activity | 1.88E-08 | 1.86E-06 | 1.65 (11216,196,3227,93) |
| GO:0038023 | signaling receptor activity | 4.28E-08 | 4.09E-06 | 1.43 (11216,395,3227,163) |
| GO:0060089 | molecular transducer activity | 4.73E-08 | 4.36E-06 | 1.42 (11216,410,3227,168) |
| GO:0004888 | transmembrane signaling receptor activity | 5.84E-08 | 5.20E-06 | 1.45 (11216,359,3227,150) |
| GO:0005230 | extracellular ligand-gated ion channel activity | 8.68E-08 | 7.46E-06 | 1.93 (11216,90,3227,50) |
| GO:0005267 | potassium channel activity | 1.48E-07 | 1.23E-05 | 2.03 (11216,72,3227,42) |
| GO:0015079 | potassium ion transmembrane transporter activity | 6.54E-07 | 5.27E-05 | 1.85 (11216,92,3227,49) |
| GO:0016773 | phosphotransferase activity, alcohol group as acceptor | 1.85E-06 | 1.44E-04 | 1.35 (11216,448,3227,174) |
| GO:0004674 | protein serine/threonine kinase activity | 2.16E-06 | 1.64E-04 | 1.44 (11216,287,3227,119) |
| GO:0099094 | ligand-gated cation channel activity | 4.83E-06 | 3.56E-04 | 2.02 (11216,55,3227,32) |
| GO:0015081 | sodium ion transmembrane transporter activity | 6.04E-06 | 4.33E-04 | 1.92 (11216,65,3227,36) |
| GO:0008237 | metallopeptidase activity | 8.08E-06 | 5.64E-04 | 1.59 (11216,149,3227,68) |
| GO:0005249 | voltage-gated potassium channel activity | 9.31E-06 | 6.32E-04 | 2.50 (11216,25,3227,18) |
| GO:0008238 | exopeptidase activity | 1.51E-05 | 9.98E-04 | 1.87 (11216,67,3227,36) |
| GO:0070011 | peptidase activity, acting on L-amino acid peptides | 2.33E-05 | 1.50E-03 | 1.36 (11216,329,3227,129) |
| GO:0022842 | narrow pore channel activity | 2.56E-05 | 1.61E-03 | 2.05 (11216,44,3227,26) |
| GO:0022841 | potassium ion leak channel activity | 2.56E-05 | 1.57E-03 | 2.05 (11216,44,3227,26) |
| GO:0022840 | leak channel activity | 2.56E-05 | 1.54E-03 | 2.05 (11216,44,3227,26) |
| GO:0008233 | peptidase activity | 2.79E-05 | 1.64E-03 | 1.35 (11216,345,3227,134) |
| GO:0022843 | voltage-gated cation channel activity | 2.80E-05 | 1.60E-03 | 2.18 (11216,35,3227,22) |
| GO:0016757 | transferase activity, transferring glycosyl groups | 2.91E-05 | 1.63E-03 | 1.42 (11216,242,3227,99) |
| GO:0004222 | metalloendopeptidase activity | 3.06E-05 | 1.68E-03 | 1.68 (11216,97,3227,47) |
| GO:0016301 | kinase activity | 5.54E-05 | 2.98E-03 | 1.27 (11216,508,3227,186) |
| GO:0008236 | serine-type peptidase activity | 8.23E-05 | 4.33E-03 | 1.86 (11216,56,3227,30) |
| GO:0017171 | serine hydrolase activity | 8.23E-05 | 4.25E-03 | 1.86 (11216,56,3227,30) |
| GO:0004713 | protein tyrosine kinase activity | 1.09E-04 | 5.53E-03 | 1.68 (11216,85,3227,41) |
| GO:0005509 | calcium ion binding | 1.21E-04 | 5.98E-03 | 1.49 (11216,154,3227,66) |
| GO:0005198 | structural molecule activity | 1.28E-04 | 6.23E-03 | 1.31 (11216,367,3227,138) |
| GO:0015280 | ligand-gated sodium channel activity | 1.57E-04 | 7.53E-03 | 2.43 (11216,20,3227,14) |
| GO:0016769 | transferase activity, transferring nitrogenous groups | 1.61E-04 | 7.57E-03 | 2.73 (11216,14,3227,11) |
| GO:0008483 | transaminase activity | 1.61E-04 | 7.44E-03 | 2.73 (11216,14,3227,11) |
| GO:0004930 | G protein-coupled receptor activity | 1.82E-04 | 8.22E-03 | 1.41 (11216,199,3227,81) |
| GO:0022804 | active transmembrane transporter activity | 2.05E-04 | 9.14E-03 | 1.42 (11216,188,3227,77) |
| GO:0022832 | voltage-gated channel activity | 2.36E-04 | 1.03E-02 | 1.89 (11216,46,3227,25) |
| GO:0005244 | voltage-gated ion channel activity | 2.36E-04 | 1.01E-02 | 1.89 (11216,46,3227,25) |
| GO:0015291 | secondary active transmembrane transporter activity | 3.14E-04 | 1.33E-02 | 1.57 (11216,102,3227,46) |
| GO:0017022 | myosin binding | 4.06E-04 | 1.69E-02 | 2.84 (11216,11,3227,9) |
| GO:0004180 | carboxypeptidase activity | 5.02E-04 | 2.06E-02 | 2.14 (11216,26,3227,16) |
| GO:0005201 | extracellular matrix structural constituent | 7.45E-04 | 3.00E-02 | 2.04 (11216,29,3227,17) |
| GO:0008188 | neuropeptide receptor activity | 8.18E-04 | 3.25E-02 | 2.26 (11216,20,3227,13) |

**(B) Downregulated genes related to the reduced cuticle structure activity**

| Genes | Fold change | Adjusted *P* value^ψ^ | Genes | Fold change | Adjusted *P* value^ψ^ |
| --- | --- | --- | --- | --- | --- |
| col-88 | 827.4 | 9.38E-05 | col-174 | 103.4 | 9.38E-05 |
| col-49 | 766.6 | 9.38E-05 | col-155 | 101.6 | 9.38E-05 |
| rol-1 | 697.1 | 9.38E-05 | rol-6 | 99.9 | 9.38E-05 |
| col-63 | 633.5 | 9.38E-05 | col-168 | 98.9 | 9.38E-05 |
| col-138 | 590.3 | 9.38E-05 | col-167 | 92.6 | 9.38E-05 |
| col-161 | 577.4 | 9.38E-05 | col-172 | 84.9 | 9.38E-05 |
| col-104 | 552.6 | 9.38E-05 | rol-8 | 80.9 | 9.38E-05 |
| col-60 | 544.3 | 9.38E-05 | col-144 | 80.3 | 9.38E-05 |
| col-77 | 531.1 | 9.38E-05 | col-159 | 77.6 | 9.38E-05 |
| col-120 | 526.2 | 9.38E-05 | col-113 | 77.3 | 9.38E-05 |
| col-162 | 475.8 | 9.38E-05 | col-150 | 67.3 | 9.38E-05 |
| col-137 | 464.1 | 7.11E-04 | bli-5 | 63 | 4.85E-03 |
| col-130 | 454.2 | 9.38E-05 | col-34 | 60.2 | 9.38E-05 |
| col-145 | 453.4 | 9.38E-05 | col-10 | 55.8 | 9.38E-05 |
| col-14 | 414.6 | 9.38E-05 | col-169 | 53.7 | 9.38E-05 |
| bli-6 | 410.6 | 9.38E-05 | col-61 | 47.2 | 9.38E-05 |
| col-91 | 393.1 | 9.38E-05 | col-81 | 41 | 9.38E-05 |
| col-12 | 352.7 | 9.38E-05 | col-89 | 34.2 | 9.38E-05 |
| col-133 | 350 | 9.38E-05 | col-54 | 33.8 | 9.38E-05 |
| col-97 | 347.2 | 9.38E-05 | col-118 | 32.8 | 9.38E-05 |
| col-71 | 334.3 | 9.38E-05 | col-147 | 32.6 | 9.38E-05 |
| col-73 | 325.1 | 9.38E-05 | col-139 | 32.5 | 9.38E-05 |
| col-13 | 311 | 9.38E-05 | col-129 | 32.1 | 9.38E-05 |
| col-109 | 305.5 | 9.38E-05 | col-111 | 31.6 | 9.38E-05 |
| col-58 | 300.7 | 9.38E-05 | col-33 | 26.8 | 6.24E-04 |
| col-65 | 298 | 9.38E-05 | col-90 | 25.9 | 9.38E-05 |
| col-79 | 284.3 | 1.04E-02 | col-186 | 23.4 | 9.38E-05 |
| col-157 | 281.6 | 9.38E-05 | sqt-3 | 22.4 | 9.38E-05 |
| col-146 | 267.8 | 9.38E-05 | col-166 | 20.3 | 9.38E-05 |
| col-154 | 248.8 | 9.38E-05 | col-69 | 19.6 | 1.39E-03 |
| col-107 | 247.4 | 9.38E-05 | col-141 | 15.6 | 9.38E-05 |
| col-156 | 239.7 | 9.38E-05 | col-149 | 14.5 | 9.38E-05 |
| col-180 | 229.2 | 9.38E-05 | col-93 | 10.2 | 1.01E-02 |
| col-38 | 213 | 9.38E-05 | col-160 | 8.1 | 9.38E-05 |
| col-7 | 213 | 9.38E-05 | col-68 | 7.2 | 9.38E-05 |
| col-173 | 208.5 | 9.38E-05 | dpy-8 | 7 | 9.38E-05 |
| col-48 | 204.2 | 9.38E-05 | col-176 | 7 | 9.38E-05 |
| col-125 | 199.6 | 9.38E-05 | col-124 | 6.5 | 9.38E-05 |
| ram-2 | 197.1 | 9.38E-05 | col-153 | 6.3 | 9.38E-05 |
| col-152 | 194.3 | 9.38E-05 | col-76 | 5.7 | 9.38E-05 |
| dpy-5 | 189.7 | 9.38E-05 | dpy-9 | 4.6 | 9.38E-05 |
| bli-2 | 186.8 | 9.38E-05 | col-19 | 3.7 | 9.38E-05 |
| col-75 | 186.7 | 3.96E-02 | col-20 | 3.6 | 9.38E-05 |
| dpy-4 | 173.3 | 9.38E-05 | col-140 | 3.6 | 9.38E-05 |
| col-39 | 158.2 | 9.38E-05 | dpy-7 | 3.5 | 9.38E-05 |
| col-110 | 154.4 | 9.38E-05 | col-181 | 2.8 | 9.38E-05 |
| lon-3 | 139.1 | 4.32E-02 | col-122 | 2.7 | 9.38E-05 |
| col-92 | 126 | 1.69E-02 | col-178 | 2.7 | 9.38E-05 |
| col-62 | 125.8 | 9.38E-05 | col-80 | 2.6 | 8.87E-03 |
| cut-4 | 123.4 | 9.38E-05 | col-184 | 2.4 | 9.38E-05 |
| sqt-2 | 117.3 | 9.38E-05 | col-142 | 2.3 | 9.38E-05 |
| sqt-1 | 116.2 | 3.82E-03 | col-179 | 2.2 | 9.38E-05 |
| cut-2 | 114.6 | 9.38E-05 | cut-5 | 2.2 | 1.85E-04 |
| col-170 | 112 | 9.38E-05 |  |  |  |

^#^ P-value is computed according to the mHG model (Eden *et al.* 2007 PLoS Comp Bio 3(3):e39). * FDR q-value is the correction of the above p-value for multiple testing using the Benjamini and Hochberg method (Benjamini and Hochberg 1995 J R Statist Soc B 57(1):289-300). ^§^ Enrichment (N, B, n, b) is defined as follows: N - total number of genes; B - total number of genes associated with a specific GO term; n - number of genes in the target set; b - number of genes in the intersection;Enrichment = (b/n) / (B/N). ^ψ^Adjusted *P* value is the correction of the P value for multiple testing using the Benjamini and Hochberg method (Benjamini and Hochberg 1995 J R Statist Soc B 57 (1):289–300).
