# Supplementary material for: The longevity response to warm temperature is neurally controlled via the regulation of collagen genes
Source: Aging Cell. 2023 Mar 9;22(5):e13815. doi: 10.1111/acel.13815 (PMC10186602; doi:10.1111/acel.13815)
Supplement: Supplementary file 13 — Table S12 [file ACEL-22-e13815-s003.docx]

**Table S12. Attenuated molecular functions in 9-day-old wild-type animals grown at 20°C relative to 1-day-old adult animals (RNA-seq analysis with 1% FDR)**

**(A) Attenuated molecular functions**

| GO term | Description | P-value^#^ | FDR q-value* | Enrichment (N, B, n, b)^§^ |
| --- | --- | --- | --- | --- |
| GO:0042302 | structural constituent of cuticle | 1.83E-28 | 4.72E-25 | 2.68 (11216,145,2911,101) |
| GO:0022857 | transmembrane transporter activity | 1.69E-22 | 2.18E-19 | 1.60 (11216,769,2911,319) |
| GO:0005215 | transporter activity | 4.96E-22 | 4.26E-19 | 1.58 (11216,804,2911,329) |
| GO:0022803 | passive transmembrane transporter activity | 4.92E-18 | 3.17E-15 | 1.87 (11216,309,2911,150) |
| GO:0015267 | channel activity | 4.92E-18 | 2.54E-15 | 1.87 (11216,309,2911,150) |
| GO:0005216 | ion channel activity | 7.88E-17 | 3.39E-14 | 1.90 (11216,267,2911,132) |
| GO:0022838 | substrate-specific channel activity | 9.75E-17 | 3.60E-14 | 1.89 (11216,273,2911,134) |
| GO:0004725 | protein tyrosine phosphatase activity | 2.29E-16 | 7.40E-14 | 2.60 (11216,89,2911,60) |
| GO:0015318 | inorganic molecular entity transmembrane transporter activity | 1.03E-15 | 2.96E-13 | 1.64 (11216,464,2911,198) |
| GO:0004721 | phosphoprotein phosphatase activity | 1.34E-15 | 3.46E-13 | 2.11 (11216,168,2911,92) |
| GO:0015075 | ion transmembrane transporter activity | 6.21E-15 | 1.46E-12 | 1.60 (11216,498,2911,207) |
| GO:0022836 | gated channel activity | 2.13E-14 | 4.59E-12 | 2.07 (11216,166,2911,89) |
| GO:0022839 | ion gated channel activity | 5.91E-14 | 1.17E-11 | 2.06 (11216,163,2911,87) |
| GO:0016791 | phosphatase activity | 6.74E-14 | 1.24E-11 | 1.85 (11216,241,2911,116) |
| GO:0005261 | cation channel activity | 8.42E-13 | 1.45E-10 | 2.01 (11216,161,2911,84) |
| GO:0042578 | phosphoric ester hydrolase activity | 9.87E-12 | 1.59E-09 | 1.72 (11216,273,2911,122) |
| GO:0008324 | cation transmembrane transporter activity | 1.40E-11 | 2.12E-09 | 1.65 (11216,324,2911,139) |
| GO:0022834 | ligand-gated channel activity | 1.79E-11 | 2.57E-09 | 2.10 (11216,121,2911,66) |
| GO:0015276 | ligand-gated ion channel activity | 1.79E-11 | 2.43E-09 | 2.10 (11216,121,2911,66) |
| GO:0046873 | metal ion transmembrane transporter activity | 6.98E-11 | 9.01E-09 | 1.80 (11216,205,2911,96) |
| GO:0022890 | inorganic cation transmembrane transporter activity | 1.39E-10 | 1.71E-08 | 1.65 (11216,294,2911,126) |
| GO:0015077 | monovalent inorganic cation transmembrane transporter activity | 2.40E-09 | 2.82E-07 | 1.75 (11216,196,2911,89) |
| GO:0140096 | catalytic activity, acting on a protein | 2.60E-09 | 2.92E-07 | 1.27 (11216,1215,2911,402) |
| GO:0030594 | neurotransmitter receptor activity | 3.52E-09 | 3.79E-07 | 2.01 (11216,109,2911,57) |
| GO:0004715 | non-membrane spanning protein tyrosine kinase activity | 5.00E-09 | 5.16E-07 | 2.63 (11216,44,2911,30) |
| GO:0005230 | extracellular ligand-gated ion channel activity | 8.67E-08 | 8.61E-06 | 2.01 (11216,90,2911,47) |
| GO:0004672 | protein kinase activity | 3.05E-07 | 2.91E-05 | 1.44 (11216,391,2911,146) |
| GO:0005267 | potassium channel activity | 3.09E-07 | 2.85E-05 | 2.09 (11216,72,2911,39) |
| GO:0015079 | potassium ion transmembrane transporter activity | 6.21E-07 | 5.52E-05 | 1.93 (11216,92,2911,46) |
| GO:0038023 | signaling receptor activity | 1.06E-06 | 9.08E-05 | 1.41 (11216,395,2911,145) |
| GO:0060089 | molecular transducer activity | 1.47E-06 | 1.22E-04 | 1.40 (11216,410,2911,149) |
| GO:0015081 | sodium ion transmembrane transporter activity | 1.49E-06 | 1.20E-04 | 2.07 (11216,65,2911,35) |
| GO:0099094 | ligand-gated cation channel activity | 1.61E-06 | 1.26E-04 | 2.17 (11216,55,2911,31) |
| GO:0004888 | transmembrane signaling receptor activity | 1.68E-06 | 1.27E-04 | 1.43 (11216,359,2911,133) |
| GO:0008238 | exopeptidase activity | 3.76E-06 | 2.77E-04 | 2.01 (11216,67,2911,35) |
| GO:0008237 | metallopeptidase activity | 4.34E-06 | 3.11E-04 | 1.65 (11216,149,2911,64) |
| GO:0005249 | voltage-gated potassium channel activity | 1.24E-05 | 8.63E-04 | 2.62 (11216,25,2911,17) |
| GO:0070011 | peptidase activity, acting on L-amino acid peptides | 2.13E-05 | 1.44E-03 | 1.39 (11216,329,2911,119) |
| GO:0022843 | voltage-gated cation channel activity | 2.13E-05 | 1.41E-03 | 2.31 (11216,35,2911,21) |
| GO:0004222 | metalloendopeptidase activity | 2.65E-05 | 1.71E-03 | 1.75 (11216,97,2911,44) |
| GO:0008233 | peptidase activity | 3.33E-05 | 2.10E-03 | 1.37 (11216,345,2911,123) |
| GO:0016773 | phosphotransferase activity, alcohol group as acceptor | 3.36E-05 | 2.06E-03 | 1.32 (11216,448,2911,154) |
| GO:0022804 | active transmembrane transporter activity | 3.42E-05 | 2.05E-03 | 1.52 (11216,188,2911,74) |
| GO:0015280 | ligand-gated sodium channel activity | 4.57E-05 | 2.68E-03 | 2.70 (11216,20,2911,14) |
| GO:0005198 | structural molecule activity | 4.61E-05 | 2.64E-03 | 1.35 (11216,367,2911,129) |
| GO:0022842 | narrow pore channel activity | 5.00E-05 | 2.81E-03 | 2.10 (11216,44,2911,24) |
| GO:0022841 | potassium ion leak channel activity | 5.00E-05 | 2.75E-03 | 2.10 (11216,44,2911,24) |
| GO:0022840 | leak channel activity | 5.00E-05 | 2.69E-03 | 2.10 (11216,44,2911,24) |
| GO:0004713 | protein tyrosine kinase activity | 5.47E-05 | 2.88E-03 | 1.77 (11216,85,2911,39) |
| GO:0016769 | transferase activity, transferring nitrogenous groups | 5.74E-05 | 2.97E-03 | 3.03 (11216,14,2911,11) |
| GO:0008483 | transaminase activity | 5.74E-05 | 2.91E-03 | 3.03 (11216,14,2911,11) |
| GO:0008236 | serine-type peptidase activity | 9.59E-05 | 4.76E-03 | 1.93 (11216,56,2911,28) |
| GO:0017171 | serine hydrolase activity | 9.59E-05 | 4.67E-03 | 1.93 (11216,56,2911,28) |
| GO:0015291 | secondary active transmembrane transporter activity | 1.16E-04 | 5.53E-03 | 1.66 (11216,102,2911,44) |
| GO:0004180 | carboxypeptidase activity | 1.37E-04 | 6.41E-03 | 2.37 (11216,26,2911,16) |
| GO:0016757 | transferase activity, transferring glycosyl groups | 1.96E-04 | 9.05E-03 | 1.40 (11216,242,2911,88) |
| GO:0005201 | extracellular matrix structural constituent | 1.96E-04 | 8.89E-03 | 2.26 (11216,29,2911,17) |

**(B) Downregulated genes related to the reduced cuticle structure activity**

| Genes | Fold change | Adjusted *P* value^ψ^ | Genes | Fold change | Adjusted *P* value^ψ^ |
| --- | --- | --- | --- | --- | --- |
| col-88 | 827.4 | 9.38E-05 | col-174 | 103.4 | 9.38E-05 |
| col-49 | 766.6 | 9.38E-05 | col-155 | 101.6 | 9.38E-05 |
| rol-1 | 697.1 | 9.38E-05 | rol-6 | 99.9 | 9.38E-05 |
| col-63 | 633.5 | 9.38E-05 | col-168 | 98.9 | 9.38E-05 |
| col-138 | 590.3 | 9.38E-05 | col-167 | 92.6 | 9.38E-05 |
| col-161 | 577.4 | 9.38E-05 | col-172 | 84.9 | 9.38E-05 |
| col-104 | 552.6 | 9.38E-05 | rol-8 | 80.9 | 9.38E-05 |
| col-60 | 544.3 | 9.38E-05 | col-144 | 80.3 | 9.38E-05 |
| col-77 | 531.1 | 9.38E-05 | col-159 | 77.6 | 9.38E-05 |
| col-120 | 526.2 | 9.38E-05 | col-113 | 77.3 | 9.38E-05 |
| col-162 | 475.8 | 9.38E-05 | col-150 | 67.3 | 9.38E-05 |
| col-137 | 464.1 | 7.11E-04 | bli-5 | 63 | 4.85E-03 |
| col-130 | 454.2 | 9.38E-05 | col-34 | 60.2 | 9.38E-05 |
| col-145 | 453.4 | 9.38E-05 | col-10 | 55.8 | 9.38E-05 |
| col-14 | 414.6 | 9.38E-05 | col-169 | 53.7 | 9.38E-05 |
| bli-6 | 410.6 | 9.38E-05 | col-61 | 47.2 | 9.38E-05 |
| col-91 | 393.1 | 9.38E-05 | col-81 | 41 | 9.38E-05 |
| col-12 | 352.7 | 9.38E-05 | col-89 | 34.2 | 9.38E-05 |
| col-133 | 350 | 9.38E-05 | col-54 | 33.8 | 9.38E-05 |
| col-97 | 347.2 | 9.38E-05 | col-118 | 32.8 | 9.38E-05 |
| col-71 | 334.3 | 9.38E-05 | col-147 | 32.6 | 9.38E-05 |
| col-73 | 325.1 | 9.38E-05 | col-139 | 32.5 | 9.38E-05 |
| col-13 | 311 | 9.38E-05 | col-129 | 32.1 | 9.38E-05 |
| col-109 | 305.5 | 9.38E-05 | col-111 | 31.6 | 9.38E-05 |
| col-58 | 300.7 | 9.38E-05 | col-33 | 26.8 | 6.24E-04 |
| col-65 | 298 | 9.38E-05 | col-90 | 25.9 | 9.38E-05 |
| col-79 | 284.3 | 1.04E-02 | col-186 | 23.4 | 9.38E-05 |
| col-157 | 281.6 | 9.38E-05 | sqt-3 | 22.4 | 9.38E-05 |
| col-146 | 267.8 | 9.38E-05 | col-166 | 20.3 | 9.38E-05 |
| col-154 | 248.8 | 9.38E-05 | col-69 | 19.6 | 1.39E-03 |
| col-107 | 247.4 | 9.38E-05 | col-141 | 15.6 | 9.38E-05 |
| col-156 | 239.7 | 9.38E-05 | col-149 | 14.5 | 9.38E-05 |
| col-180 | 229.2 | 9.38E-05 | col-93 | 10.2 | 1.01E-02 |
| col-38 | 213 | 9.38E-05 | col-160 | 8.1 | 9.38E-05 |
| col-7 | 213 | 9.38E-05 | col-68 | 7.2 | 9.38E-05 |
| col-173 | 208.5 | 9.38E-05 | dpy-8 | 7 | 9.38E-05 |
| col-48 | 204.2 | 9.38E-05 | col-176 | 7 | 9.38E-05 |
| col-125 | 199.6 | 9.38E-05 | col-124 | 6.5 | 9.38E-05 |
| ram-2 | 197.1 | 9.38E-05 | col-153 | 6.3 | 9.38E-05 |
| col-152 | 194.3 | 9.38E-05 | col-76 | 5.7 | 9.38E-05 |
| dpy-5 | 189.7 | 9.38E-05 | dpy-9 | 4.6 | 9.38E-05 |
| bli-2 | 186.8 | 9.38E-05 | col-19 | 3.7 | 9.38E-05 |
| col-75 | 186.7 | 3.96E-02 | col-20 | 3.6 | 9.38E-05 |
| dpy-4 | 173.3 | 9.38E-05 | col-140 | 3.6 | 9.38E-05 |
| col-39 | 158.2 | 9.38E-05 | dpy-7 | 3.5 | 9.38E-05 |
| col-110 | 154.4 | 9.38E-05 | col-181 | 2.8 | 9.38E-05 |
| lon-3 | 139.1 | 4.32E-02 | col-122 | 2.7 | 9.38E-05 |
| col-92 | 126 | 1.69E-02 | col-178 | 2.7 | 9.38E-05 |
| col-62 | 125.8 | 9.38E-05 | col-80 | 2.6 | 8.87E-03 |
| cut-4 | 123.4 | 9.38E-05 | col-184 | 2.4 | 9.38E-05 |
| sqt-2 | 117.3 | 9.38E-05 | col-142 | 2.3 | 9.38E-05 |
| sqt-1 | 116.2 | 3.82E-03 | col-179 | 2.2 | 9.38E-05 |
| cut-2 | 114.6 | 9.38E-05 | cut-5 | 2.2 | 1.85E-04 |
| col-170 | 112 | 9.38E-05 |  |  |  |

^#^ P-value is computed according to the mHG model (Eden *et al.* 2007 PLoS Comp Bio 3(3):e39). * FDR q-value is the correction of the above p-value for multiple testing using the Benjamini and Hochberg method (Benjamini and Hochberg 1995 J R Statist Soc B 57(1):289-300). ^§^ Enrichment (N, B, n, b) is defined as follows: N - total number of genes; B - total number of genes associated with a specific GO term; n - number of genes in the target set; b - number of genes in the intersection;Enrichment = (b/n) / (B/N). ^ψ^Adjusted *P* value is the correction of the P value for multiple testing using the Benjamini and Hochberg method (Benjamini and Hochberg 1995 J R Statist Soc B 57 (1):289–300).
