# Supplementary material for: The longevity response to warm temperature is neurally controlled via the regulation of collagen genes
Source: Aging Cell. 2023 Mar 9;22(5):e13815. doi: 10.1111/acel.13815 (PMC10186602; doi:10.1111/acel.13815)
Supplement: Supplementary file 15 — Table S14 [file ACEL-22-e13815-s020.docx]

**Table S14. Attenuated molecular functions in 1-day-old wild-type adults grown at 25°C relative to those grown at 20°C (RNA-seq analysis with 1% FDR)**

**(A) Attenuated molecular functions**

| GO term | Description | P-value^#^ | FDR q-value* | Enrichment (N, B, n, b)^§^ |
| --- | --- | --- | --- | --- |
| GO:0005216 | ion channel activity | 1.02E-20 | 2.62E-17 | 2.00 (10856,257,2955,140) |
| GO:0022803 | passive transmembrane transporter activity | 1.18E-20 | 1.52E-17 | 1.92 (10856,298,2955,156) |
| GO:0015267 | channel activity | 1.18E-20 | 1.01E-17 | 1.92 (10856,298,2955,156) |
| GO:0022838 | substrate-specific channel activity | 5.22E-20 | 3.37E-17 | 1.97 (10856,263,2955,141) |
| GO:0042302 | structural constituent of cuticle | 1.69E-19 | 8.71E-17 | 2.34 (10856,140,2955,89) |
| GO:0004888 | transmembrane signaling receptor activity | 2.58E-19 | 1.11E-16 | 1.84 (10856,329,2955,165) |
| GO:0022836 | gated channel activity | 1.43E-18 | 5.28E-16 | 2.22 (10856,157,2955,95) |
| GO:0004725 | protein tyrosine phosphatase activity | 1.64E-18 | 5.28E-16 | 2.64 (10856,89,2955,64) |
| GO:0022839 | ion gated channel activity | 4.00E-18 | 1.15E-15 | 2.22 (10856,154,2955,93) |
| GO:0004721 | phosphoprotein phosphatase activity | 1.45E-17 | 3.75E-15 | 2.14 (10856,168,2955,98) |
| GO:0038023 | signaling receptor activity | 4.42E-17 | 1.04E-14 | 1.75 (10856,362,2955,172) |
| GO:0060089 | molecular transducer activity | 4.03E-16 | 8.65E-14 | 1.71 (10856,377,2955,175) |
| GO:0005261 | cation channel activity | 1.00E-15 | 1.99E-13 | 2.12 (10856,154,2955,89) |
| GO:0022834 | ligand-gated channel activity | 8.96E-15 | 1.65E-12 | 2.28 (10856,113,2955,70) |
| GO:0015276 | ligand-gated ion channel activity | 8.96E-15 | 1.54E-12 | 2.28 (10856,113,2955,70) |
| GO:0016791 | phosphatase activity | 1.53E-13 | 2.47E-11 | 1.81 (10856,239,2955,118) |
| GO:0022857 | transmembrane transporter activity | 3.32E-13 | 5.03E-11 | 1.43 (10856,750,2955,292) |
| GO:0005215 | transporter activity | 3.97E-13 | 5.69E-11 | 1.42 (10856,785,2955,303) |
| GO:0015318 | inorganic molecular entity transmembrane transporter activity | 5.30E-13 | 7.19E-11 | 1.56 (10856,451,2955,192) |
| GO:0004672 | protein kinase activity | 1.30E-12 | 1.68E-10 | 1.61 (10856,382,2955,167) |
| GO:0030594 | neurotransmitter receptor activity | 1.56E-12 | 1.91E-10 | 2.20 (10856,105,2955,63) |
| GO:0015075 | ion transmembrane transporter activity | 2.35E-11 | 2.76E-09 | 1.50 (10856,485,2955,198) |
| GO:0042578 | phosphoric ester hydrolase activity | 2.94E-11 | 3.30E-09 | 1.68 (10856,271,2955,124) |
| GO:0005230 | extracellular ligand-gated ion channel activity | 9.23E-11 | 9.91E-09 | 2.22 (10856,86,2955,52) |
| GO:0004715 | non-membrane spanning protein tyrosine kinase activity | 1.49E-10 | 1.54E-08 | 2.73 (10856,43,2955,32) |
| GO:0004930 | G protein-coupled receptor activity | 1.77E-10 | 1.76E-08 | 1.82 (10856,178,2955,88) |
| GO:0140096 | catalytic activity, acting on a protein | 2.24E-10 | 2.14E-08 | 1.29 (10856,1194,2955,418) |
| GO:0099094 | ligand-gated cation channel activity | 2.04E-09 | 1.88E-07 | 2.50 (10856,50,2955,34) |
| GO:0016773 | phosphotransferase activity, alcohol group as acceptor | 1.55E-08 | 1.38E-06 | 1.44 (10856,438,2955,172) |
| GO:0046873 | metal ion transmembrane transporter activity | 1.68E-08 | 1.44E-06 | 1.68 (10856,197,2955,90) |
| GO:0005267 | potassium channel activity | 5.72E-08 | 4.76E-06 | 2.12 (10856,71,2955,41) |
| GO:0022890 | inorganic cation transmembrane transporter activity | 9.01E-08 | 7.26E-06 | 1.53 (10856,284,2955,118) |
| GO:0008324 | cation transmembrane transporter activity | 9.63E-08 | 7.52E-06 | 1.50 (10856,314,2955,128) |
| GO:0004713 | protein tyrosine kinase activity | 1.06E-07 | 8.03E-06 | 2.02 (10856,82,2955,45) |
| GO:0015079 | potassium ion transmembrane transporter activity | 1.31E-07 | 9.66E-06 | 1.96 (10856,90,2955,48) |
| GO:0004674 | protein serine/threonine kinase activity | 2.18E-07 | 1.56E-05 | 1.51 (10856,282,2955,116) |
| GO:0015077 | monovalent inorganic cation transmembrane transporter activity | 1.70E-06 | 1.19E-04 | 1.58 (10856,188,2955,81) |
| GO:0022843 | voltage-gated cation channel activity | 2.53E-06 | 1.72E-04 | 2.45 (10856,33,2955,22) |
| GO:0016301 | kinase activity | 5.10E-06 | 3.37E-04 | 1.33 (10856,498,2955,180) |
| GO:0015081 | sodium ion transmembrane transporter activity | 1.01E-05 | 6.50E-04 | 1.99 (10856,59,2955,32) |
| GO:0022824 | transmitter-gated ion channel activity | 2.13E-05 | 1.34E-03 | 2.45 (10856,27,2955,18) |
| GO:0022835 | transmitter-gated channel activity | 2.13E-05 | 1.31E-03 | 2.45 (10856,27,2955,18) |
| GO:0005201 | extracellular matrix structural constituent | 2.13E-05 | 1.28E-03 | 2.45 (10856,27,2955,18) |
| GO:0022832 | voltage-gated channel activity | 3.28E-05 | 1.92E-03 | 2.09 (10856,44,2955,25) |
| GO:0005244 | voltage-gated ion channel activity | 3.28E-05 | 1.88E-03 | 2.09 (10856,44,2955,25) |
| GO:0015280 | ligand-gated sodium channel activity | 3.30E-05 | 1.85E-03 | 2.81 (10856,17,2955,13) |
| GO:0005249 | voltage-gated potassium channel activity | 6.21E-05 | 3.41E-03 | 2.45 (10856,24,2955,16) |
| GO:0005272 | sodium channel activity | 7.18E-05 | 3.86E-03 | 2.50 (10856,22,2955,15) |
| GO:0005102 | signaling receptor binding | 9.61E-05 | 5.05E-03 | 1.45 (10856,200,2955,79) |
| GO:0022842 | narrow pore channel activity | 1.14E-04 | 5.89E-03 | 2.00 (10856,44,2955,24) |
| GO:0022841 | potassium ion leak channel activity | 1.14E-04 | 5.77E-03 | 2.00 (10856,44,2955,24) |
| GO:0022840 | leak channel activity | 1.14E-04 | 5.66E-03 | 2.00 (10856,44,2955,24) |
| GO:0005509 | calcium ion binding | 1.48E-04 | 7.21E-03 | 1.51 (10856,151,2955,62) |
| GO:0004722 | protein serine/threonine phosphatase activity | 1.55E-04 | 7.39E-03 | 1.76 (10856,71,2955,34) |

**(B) Downregulated genes related to the reduced cuticle structure activity**

| Genes | Fold change | Adjusted *P* value^ψ^ | Genes | Fold change | Adjusted *P* value^ψ^ |
| --- | --- | --- | --- | --- | --- |
| col-88 | 2341.3 | 9.74E-05 | col-146 | 239.8 | 9.74E-05 |
| col-49 | 1800.4 | 9.74E-05 | col-12 | 200.8 | 9.74E-05 |
| rol-1 | 1672.2 | 9.74E-05 | col-38 | 186.1 | 9.74E-05 |
| col-161 | 1505.4 | 9.74E-05 | col-110 | 170.8 | 9.74E-05 |
| cut-2 | 1377.2 | 9.74E-05 | col-169 | 165.9 | 9.74E-05 |
| col-63 | 1318.0 | 9.74E-05 | col-180 | 162.8 | 9.74E-05 |
| col-156 | 1299.9 | 9.74E-05 | col-7 | 151.6 | 9.74E-05 |
| col-120 | 1229.9 | 9.74E-05 | bli-6 | 150.3 | 9.74E-05 |
| col-138 | 1172.0 | 9.74E-05 | col-89 | 141.7 | 9.74E-05 |
| col-73 | 1124.8 | 9.74E-05 | col-111 | 107.1 | 9.74E-05 |
| col-91 | 1089.5 | 9.74E-05 | bli-1 | 97.2 | 9.74E-05 |
| col-137 | 1066.7 | 0.003494 | col-113 | 87.7 | 9.74E-05 |
| dpy-5 | 1002.6 | 9.74E-05 | col-62 | 70.9 | 9.74E-05 |
| col-77 | 974.0 | 9.74E-05 | col-61 | 64.0 | 9.74E-05 |
| col-145 | 963.3 | 9.74E-05 | col-13 | 60.9 | 9.74E-05 |
| col-109 | 926.0 | 9.74E-05 | sqt-3 | 48.8 | 9.74E-05 |
| col-65 | 920.6 | 9.74E-05 | ram-2 | 42.6 | 9.74E-05 |
| col-60 | 843.2 | 9.74E-05 | col-144 | 37.8 | 9.74E-05 |
| dpy-13 | 823.2 | 9.74E-05 | col-186 | 37.3 | 9.74E-05 |
| sqt-2 | 812.6 | 9.74E-05 | col-10 | 33.3 | 9.74E-05 |
| col-14 | 811.7 | 9.74E-05 | cut-4 | 32.8 | 9.74E-05 |
| col-162 | 787.5 | 9.74E-05 | col-69 | 23.7 | 0.001077 |
| col-97 | 779.7 | 9.74E-05 | dpy-8 | 22.0 | 9.74E-05 |
| col-170 | 777.2 | 9.74E-05 | col-150 | 18.1 | 9.74E-05 |
| sqt-1 | 735.1 | 0.002925 | col-34 | 16.3 | 0.001503 |
| col-157 | 706.7 | 9.74E-05 | bli-5 | 15.7 | 0.000643 |
| col-107 | 693.9 | 9.74E-05 | dpy-7 | 15.6 | 9.74E-05 |
| col-154 | 670.5 | 9.74E-05 | col-147 | 13.8 | 9.74E-05 |
| col-130 | 657.6 | 9.74E-05 | col-68 | 12.1 | 9.74E-05 |
| dpy-4 | 624.1 | 9.74E-05 | cut-5 | 11.3 | 9.74E-05 |
| col-104 | 617.0 | 9.74E-05 | col-159 | 11.0 | 9.74E-05 |
| rol-6 | 540.3 | 9.74E-05 | col-166 | 9.4 | 9.74E-05 |
| col-71 | 446.1 | 9.74E-05 | col-155 | 8.8 | 9.74E-05 |
| col-58 | 411.6 | 9.74E-05 | col-149 | 6.9 | 9.74E-05 |
| col-133 | 406.4 | 9.74E-05 | col-118 | 6.2 | 9.74E-05 |
| col-125 | 397.6 | 9.74E-05 | dpy-9 | 5.9 | 9.74E-05 |
| col-172 | 395.3 | 0.004226 | col-139 | 5.6 | 9.74E-05 |
| col-79 | 340.7 | 9.74E-05 | dpy-2 | 5.2 | 9.74E-05 |
| rol-8 | 339.6 | 9.74E-05 | col-129 | 4.2 | 9.74E-05 |
| col-90 | 337.2 | 9.74E-05 | col-99 | 3.1 | 9.74E-05 |
| col-167 | 316.9 | 9.74E-05 | col-76 | 3.0 | 9.74E-05 |
| col-168 | 309.3 | 9.74E-05 | col-141 | 2.9 | 9.74E-05 |
| col-48 | 279.6 | 9.74E-05 | col-64 | 2.8 | 9.74E-05 |
| col-173 | 279.4 | 9.74E-05 | col-153 | 2.0 | 9.74E-05 |
| col-174 | 260.1 | 9.74E-05 |  |  |  |

^#^ P-value is computed according to the mHG model (Eden *et al.* 2007 PLoS Comp Bio 3(3):e39). * FDR q-value is the correction of the above p-value for multiple testing using the Benjamini and Hochberg method (Benjamini and Hochberg 1995 J R Statist Soc B 57(1):289-300). ^§^ Enrichment (N, B, n, b) is defined as follows: N - total number of genes; B - total number of genes associated with a specific GO term; n - number of genes in the target set; b - number of genes in the intersection;Enrichment = (b/n) / (B/N). ^ψ^Adjusted *P* value is the correction of the P value for multiple testing using the Benjamini and Hochberg method (Benjamini and Hochberg 1995 J R Statist Soc B 57 (1):289–300).
