# Supplementary material for: The longevity response to warm temperature is neurally controlled via the regulation of collagen genes
Source: Aging Cell. 2023 Mar 9;22(5):e13815. doi: 10.1111/acel.13815 (PMC10186602; doi:10.1111/acel.13815)
Supplement: Supplementary file 16 — Table S15 [file ACEL-22-e13815-s001.docx]

**Table S15. Enriched molecular functions in 1-day-old adult *npr-8(ok1439)* animals grown at 20°C relative to wild-type animals**

**(A) Enriched molecular functions**

| GO term | Description | P-value^#^ | FDR q-value* | Enrichment (N, B, n, b)^§^ |
| --- | --- | --- | --- | --- |
| GO:0042302 | structural constituent of cuticle | 3.08E-06 | 7.93E-03 | 3.91 (10882,139,320,16) |
| GO:0004867 | serine-type endopeptidase inhibitor activity | 1.79E-05 | 2.30E-02 | 5.23 (10882,65,320,10) |
| GO:0008061 | chitin binding | 3.43E-05 | 2.94E-02 | 12.15 (10882,14,320,5) |
| GO:0004866 | endopeptidase inhibitor activity | 6.39E-05 | 4.11E-02 | 4.53 (10882,75,320,10) |
| GO:0030246 | carbohydrate binding | 8.95E-05 | 4.61E-02 | 2.88 (10882,201,320,17) |
| GO:0061135 | endopeptidase regulator activity | 8.97E-05 | 3.85E-02 | 4.36 (10882,78,320,10) |
| GO:0030414 | peptidase inhibitor activity | 1.11E-04 | 4.10E-02 | 4.25 (10882,80,320,10) |

**(B) Upregulated genes related to the enriched cuticle structure activity**

| Genes | Fold change | Adjusted *P* value^ψ^ | Genes | Fold change | Adjusted *P* value^ψ^ |
| --- | --- | --- | --- | --- | --- |
| col-98 | 10.7 | 2.95E-02 | col-176 | 5.6 | 4.91E-04 |
| col-143 | 8.6 | 4.91E-04 | let-653 | 4.9 | 4.91E-04 |
| dpy-8 | 7.5 | 4.91E-04 | col-101 | 3.9 | 4.91E-04 |
| dpy-2 | 7.1 | 4.91E-04 | col-41 | 3.9 | 4.91E-04 |
| dpy-7 | 6.9 | 4.91E-04 | bli-1 | 3.7 | 3.05E-02 |
| dpy-9 | 6.7 | 4.91E-04 | col-149 | 3.6 | 4.91E-04 |
| Y69H2.14 | 6 | 4.91E-04 | col-142 | 3.2 | 4.91E-04 |
| col-103 | 5.9 | 4.91E-04 | col-147 | 2.2 | 4.91E-04 |

^#^ P-value is computed according to the mHG model (Eden *et al.* 2007 PLoS Comp Bio 3(3):e39). * FDR q-value is the correction of the above p-value for multiple testing using the Benjamini and Hochberg method (Benjamini and Hochberg 1995 J R Statist Soc B 57(1):289-300). ^§^ Enrichment (N, B, n, b) is defined as follows: N - total number of genes; B - total number of genes associated with a specific GO term; n - number of genes in the target set; b - number of genes in the intersection;Enrichment = (b/n) / (B/N). ^ψ^Adjusted *P* value is the correction of the P value for multiple testing using the Benjamini and Hochberg method (Benjamini and Hochberg 1995 J R Statist Soc B 57 (1):289–300).
