# Supplementary material for: The longevity response to warm temperature is neurally controlled via the regulation of collagen genes
Source: Aging Cell. 2023 Mar 9;22(5):e13815. doi: 10.1111/acel.13815 (PMC10186602; doi:10.1111/acel.13815)
Supplement: Supplementary file 21 — Table S20 [file ACEL-22-e13815-s017.docx]

**Table S20. Enriched molecular functions in *npr-8(ok1439)* animals relative to wild-type animals at 25°C but not at 20°C in Day 1**

**(A) Enriched molecular functions**

| GO term | Description | P-value^#^ | FDR q-value* | Enrichment (N, B, n, b)^§^ |
| --- | --- | --- | --- | --- |
| GO:0042302 | structural constituent of cuticle | 5.11E-39 | 1.30E-35 | 6.53 (9627,122,785,65) |
| GO:0004721 | phosphoprotein phosphatase activity | 3.48E-27 | 4.43E-24 | 4.96 (9627,146,785,59) |
| GO:0004725 | protein tyrosine phosphatase activity | 1.16E-24 | 9.86E-22 | 6.45 (9627,78,785,41) |
| GO:0140096 | catalytic activity, acting on a protein | 9.05E-20 | 5.76E-17 | 1.97 (9627,1066,785,171) |
| GO:0004672 | protein kinase activity | 7.21E-19 | 3.67E-16 | 2.89 (9627,340,785,80) |
| GO:0016791 | phosphatase activity | 7.95E-19 | 3.37E-16 | 3.48 (9627,215,785,61) |
| GO:0042578 | phosphoric ester hydrolase activity | 7.79E-16 | 2.83E-13 | 3.05 (9627,245,785,61) |
| GO:0004715 | non-membrane spanning protein tyrosine kinase activity | 1.57E-15 | 5.00E-13 | 7.05 (9627,40,785,23) |
| GO:0016773 | phosphotransferase activity, alcohol group as acceptor | 2.37E-15 | 6.69E-13 | 2.51 (9627,395,785,81) |
| GO:0016301 | kinase activity | 1.66E-12 | 4.21E-10 | 2.23 (9627,451,785,82) |
| GO:0005198 | structural molecule activity | 3.28E-12 | 7.60E-10 | 2.44 (9627,337,785,67) |
| GO:0004674 | protein serine/threonine kinase activity | 1.88E-11 | 3.99E-09 | 2.61 (9627,258,785,55) |
| GO:0004713 | protein tyrosine kinase activity | 1.12E-10 | 2.19E-08 | 4.32 (9627,71,785,25) |
| GO:0016788 | hydrolase activity, acting on ester bonds | 5.01E-09 | 9.10E-07 | 1.97 (9627,474,785,76) |
| GO:0016772 | transferase activity, transferring phosphorus-containing groups | 2.62E-08 | 4.45E-06 | 1.84 (9627,546,785,82) |
| GO:0004722 | protein serine/threonine phosphatase activity | 4.57E-06 | 7.27E-04 | 3.42 (9627,61,785,17) |
| GO:0005102 | signaling receptor binding | 2.49E-05 | 3.72E-03 | 2.16 (9627,182,785,32) |

**(B) Upregulated genes related to the enriched cuticle structure activity**

| Gene | Fold change | Adjusted *P* value^ψ^ | Gene | Fold change | Adjusted *P* value^ψ^ |
| --- | --- | --- | --- | --- | --- |
| col-7 | 36.1 | 2.07E-04 | col-90 | 12.2 | 2.07E-04 |
| col-88 | 27 | 2.07E-04 | col-162 | 12 | 2.07E-04 |
| cut-2 | 24.2 | 2.07E-04 | rol-6 | 11.9 | 2.07E-04 |
| col-146 | 23.8 | 2.07E-04 | col-14 | 11.8 | 2.07E-04 |
| col-170 | 22.8 | 5.67E-04 | col-97 | 11.8 | 3.85E-03 |
| col-161 | 21.7 | 2.07E-04 | col-137 | 11.4 | 2.80E-02 |
| rol-1 | 21.7 | 3.09E-02 | col-125 | 11.1 | 2.07E-04 |
| col-133 | 21.4 | 2.07E-04 | col-130 | 11.1 | 2.07E-04 |
| col-49 | 21.3 | 2.07E-04 | col-60 | 10.9 | 2.07E-04 |
| col-63 | 19.7 | 1.95E-03 | col-180 | 10.8 | 2.07E-04 |
| col-145 | 19.2 | 2.07E-04 | col-169 | 10.7 | 2.07E-04 |
| col-156 | 18.3 | 5.67E-04 | col-58 | 9.6 | 2.07E-04 |
| col-154 | 17.6 | 2.07E-04 | col-79 | 8.6 | 1.72E-02 |
| col-91 | 17.6 | 3.93E-04 | rol-8 | 8.2 | 2.07E-04 |
| col-73 | 17.5 | 2.07E-04 | col-104 | 8.2 | 1.43E-02 |
| col-167 | 17.3 | 2.07E-04 | col-13 | 8.2 | 2.07E-04 |
| col-172 | 16.6 | 2.41E-02 | col-139 | 6.4 | 2.07E-04 |
| sqt-2 | 16.6 | 2.07E-04 | col-173 | 5.3 | 2.07E-04 |
| dpy-5 | 16 | 2.07E-04 | col-129 | 5.2 | 2.07E-04 |
| col-109 | 15.8 | 2.79E-03 | bli-6 | 4.8 | 2.07E-04 |
| col-168 | 15.6 | 2.07E-04 | col-110 | 4.6 | 2.07E-04 |
| col-77 | 15.5 | 2.07E-04 | col-150 | 4.5 | 2.07E-04 |
| sqt-1 | 15.4 | 3.20E-03 | col-89 | 4.1 | 2.07E-04 |
| col-62 | 15.2 | 2.07E-04 | col-159 | 4 | 2.07E-04 |
| dpy-13 | 15.1 | 2.79E-03 | col-141 | 3.5 | 2.07E-04 |
| col-138 | 15 | 2.07E-04 | col-113 | 3.1 | 2.07E-04 |
| col-12 | 14.4 | 2.07E-04 | col-10 | 3.1 | 2.07E-04 |
| lon-3 | 14.4 | 1.77E-02 | col-144 | 3 | 2.07E-04 |
| col-65 | 14 | 2.07E-04 | col-38 | 3 | 2.07E-04 |
| dpy-4 | 13.1 | 2.07E-04 | col-111 | 2.5 | 2.07E-04 |
| col-120 | 13.1 | 2.07E-04 | cut-4 | 2.4 | 2.07E-04 |
| col-107 | 12.7 | 1.05E-03 | col-166 | 2 | 2.07E-04 |
| col-157 | 12.6 | 2.07E-04 |  |  |  |

^#^ P-value is computed according to the mHG model (Eden *et al.* 2007 PLoS Comp Bio 3(3):e39). * FDR q-value is the correction of the above p-value for multiple testing using the Benjamini and Hochberg method (Benjamini and Hochberg 1995 J R Statist Soc B 57(1):289-300). ^§^ Enrichment (N, B, n, b) is defined as follows: N - total number of genes; B - total number of genes associated with a specific GO term; n - number of genes in the target set; b - number of genes in the intersection;Enrichment = (b/n) / (B/N). ^ψ^Adjusted *P* value is the correction of the P value for multiple testing using the Benjamini and Hochberg method (Benjamini and Hochberg 1995 J R Statist Soc B 57 (1):289–300).
